# Empirical treatment against cytomegalovirus and tuberculosis in HIV-infected infants with severe pneumonia: study protocol for a multicenter, open-label randomized controlled clinical trial

**DOI:** 10.1186/s13063-022-06203-1

**Published:** 2022-06-27

**Authors:** Pablo Rojo, Cinta Moraleda, Alfredo Tagarro, Sara Domínguez-Rodríguez, Lola Madrid Castillo, Luis Manuel Prieto Tato, Aranzazu Sancho López, Lilit Manukyan, Olivier Marcy, Valeriane Leroy, Alessandra Nardone, David Burger, Quique Bassat, Matthew Bates, Raoul Moh, Pui-Ying Iroh Tam, Tisungane Mvalo, Justina Magallhaes, W. Chris Buck, Jahit Sacarlal, Victor Musiime, Chishala Chabala, Hilda Angela Mujuru

**Affiliations:** 1 Servicio de Pediatria. Hospital Universitario 12 de Octubre, Servicio Madrileño de Salud (SERMAS), Madrid, Spain; 2 Unidad Pediátrica de Investigación y Ensayos Clínicos (UPIC). Instituto de Investigación Sanitaria Hospital 12 de Octubre (i+12), Fundación Biomedica del Hospital Universitario 12 de Octubre (FIB-H12O), Madrid, Spain; 3 Servicio de Pediatria. Hospital Universitario Infanta Sofia, Servicio Madrileño de Salud (SERMAS), Madrid, Spain; 4 Facultad de Ciencias Biomédicas, Universidad Europea de Madrid., Madrid, Spain; 5 London School of Hygiene & Tropical Medicine (LMC), London, UK; 6 Pharmacology Unit, Hospital Puerta de Hierro, Servicio, Madrileño de Salud (SERMAS), Madrid, Spain; 7 Université de Bordeaux, Inserm U1219, IRD EMR271, Bordeaux Population Health, GHiGS, Bordeaux, France; 8 Institut National de la Santé et de la Recherche Médicale (Inserm), University Toulouse 3,CERPOP, Toulouse, France; 9 Penta-Onlus Foundation (PENTA), Padua, Italy; 10 Stichting Katholieke Universiteit- Radboudumc (RUMC), Nijmegen, The Netherlands; 11 ISGlobal, Hospital Clínic, Universitat de Barcelona, Barcelona, Spain, Barcelona, Spain; 12 Pediatrics Department, Hospital Sant Joan de Déu, I, Universitat de Barcelona, Barcelona, Spain; 13 ICREA, Pg. Lluís Companys 23, 08010, Barcelona, Spain; 14 Centro de Investigação em Saúde de Manhiça (CISM), Maputo, Mozambique; 15 Consorcio de Investigación Biomédica en Red de Epidemiología y Salud Pública (CIBERESP), Instituto de Salud Carlos III, Madrid, Spain; 16 University of Lincoln, Lincoln, United Kingdom; 17 Unité Pédagogique de Dermatologie et Infectiologie, UFR Sciences Médicales, Programme PAC-CI, Ivory Coast, Abidjan, Côte d’Ivoire; 18 Kamuzu University Health Sciences, Blantyre, Malawi; 19 Malawi-Liverpool Wellcome Programme (MLW), Liverpool School of Tropical Medicine (LSTM), Blantyre, Malawi; 20 Lilongwe Medical Relief Trust (LMRFT), UNC Project Malawi, Lilongwe, Malawi; 21 University of California Los Angeles David Geffen School of Medicine, Los Angeles, USA; 22 Universidade Eduardo Mondlane (UEM), Maputo, Mozambique; 23 Department of Microbiology, Faculty of Medicine, Universidade Eduardo Mondlane, Maputo, Mozambique; 24 Department of Paediatrics and Child Health, College of Health Sciences, Makerere University, Kampala, Uganda; 25 Joint Clinical Research Centre, Kampala, Uganda; 26 School of Medicine, University of Zambia, Lusaka, Zambia; 27 University of Zimbabwe, Harare, Zimbabwe

**Keywords:** Cytomegalovirus, Tuberculosis, Valganciclovir, Factorial, Empirical, Pneumonia, HIV, Infants, Child mortality, Factorial randomized clinical trial

## Abstract

**Background:**

Pneumonia is the primary cause of death among HIV-infected children in Africa, with mortality rates as high as 35–40% in infants hospitalized with severe pneumonia. Bacterial pathogens and *Pneumocystis jirovecii* are well known causes of pneumonia-related death, but other important causes such as cytomegalovirus (CMV) and tuberculosis (TB) remain under-recognized and undertreated.

The immune response elicited by CMV may be associated with the risk of developing TB and TB disease progression, and CMV may accelerate disease caused both by HIV and TB. Minimally invasive autopsies confirm that CMV and TB are unrecognized causes of death in children with HIV. CMV and TB may also co-infect the same child.

The aim of this study is to compare the impact on 15-day and 1-year mortality of empirical treatment against TB and CMV plus standard of care (SoC) versus SoC in HIV-infected infants with severe pneumonia.

**Methods:**

This is a Phase II-III, open-label randomized factorial (2 × 2) clinical trial, conducted in six African countries. The trial has four arms. Infants from 28 to 365 days of age HIV-infected and hospitalized with severe pneumonia will be randomized (1:1:1:1) to (i) SoC, (ii) valganciclovir, (iii) TB-T, and (iv) TB-T plus valganciclovir. The primary endpoint of the study is all-cause mortality, focusing on the short-term (up to 15 days) and long-term (up to 1 year) mortality. Secondary endpoints include repeat hospitalization, duration of oxygen therapy during initial admission, severe and notable adverse events, adverse reactions, CMV and TB prevalence at enrolment, TB incidence, CMV viral load reduction, and evaluation of diagnostic tests such as GeneXpert Ultra on fecal and nasopharyngeal aspirate samples and urine TB-LAM.

**Discussion:**

Given the challenges in diagnosing CMV and TB in children and results from previous autopsy studies that show high rates of poly-infection in HIV-infected infants with respiratory disease, this study aims to evaluate a new approach including empirical treatment of CMV and TB for this patient population. The potential downsides of empirical treatment of these conditions include toxicity and medication interactions, which will be evaluated with pharmacokinetics sub-studies.

**Trial registration:**

ClinicalTrials.gov, NCT03915366, Universal Trial Number U111-1231-4736, Pan African Clinical Trial Registry PACTR201994797961340.

**Supplementary Information:**

The online version contains supplementary material available at 10.1186/s13063-022-06203-1.

## Administrative information

Note: the numbers in curly brackets in this protocol refer to SPIRIT checklist item numbers. The order of the items has been modified to group similar items (see http://www.equator-network.org/reporting-guidelines/spirit-2013-statement-defining-standard-protocol-items-for-clinical-trials/).

**Table:** 

Title {1}	“Empirical treatment against cytomegalovirus and tuberculosis in HIV-infected infants with severe pneumonia: study protocol for a multicenter, open-label, randomized controlled clinical trial”
Trial registration {2a and 2b}.	ClinicalTrials.gov, NCT03915366, Universal Trial Number U111-1231-4736, Pan African Clinical Trial Registry PACTR201994797961340, Eudra CT 2019-001749-42.
Protocol version {3}	2.0, January 2021
Funding {4}	EDCTP (Grant number: RIA2017MC-2013 EMPIRICAL): 7.680.618,75 €
Author details {5a}	SPIRIT guidance: Affiliations of protocol contributors. Chief Investigator, Pablo Rojo, MD, PhD, Pediatrician, Associate Professor Servicio Madrileño de Salud (SERMAS), Instituto de Investigación Sanitaria Hospital 12 de Octubre (imas12), Fundación Biomedica del Hospital Universitario 12 de Octubre (FBHU12O). Sección de Enfermedades Infecciosas Pediátricas. Servicio de Pediatría, Hospital Universitario 12 de Octubre. Unidad Pediátrica de Investigación y Ensayos Clínicos (UPIC). Scientific Coordinator, Cinta Moraleda, MD, PhD, Pediatrician Servicio Madrileño de Salud (SERMAS), Instituto de Investigación Sanitaria Hospital 12 de Octubre (imas12), Fundación Biomedica del Hospital Universitario 12 de Octubre (FBHU12O). Sección de Enfermedades Infecciosas Pediátricas. Servicio de Pediatría, Hospital Universitario 12 de Octubre. Unidad Pediátrica de Investigación y Ensayos Clínicos (UPIC). Trial Coordinator, Alfredo Tagarro, MD, PhD, Pediatrician, Senior Associate Professor. Sanitaria Hospital 12 de Octubre (imas12), Fundación Biomedica del Hospital Universitario 12 de Octubre (FBHU12O). Sección de Enfermedades Infecciosas Pediátricas. Servicio de Pediatría, Hospital Universitario 12 de Octubre. Unidad Pediátrica de Investigación y Ensayos Clínicos (UPIC). Associate Professor, Universidad Europea. Madrid, Spain Safety Coordinator, Lola Madrid, MD, PhD, Pediatrician Instituto de Investigación Sanitaria Hospital 12 de Octubre (imas12), Fundación Biomedica del Hospital Universitario 12 de Octubre (FBHU12O). Sección de Enfermedades Infecciosas Pediátricas. Servicio de Pediatría, Hospital Universitario 12 de Octubre. Unidad Pediátrica de Investigación y Ensayos Clínicos (UPIC). Assistant Professor, London School of Hygiene & Tropical Medicine, London, UK. Data Scientist, Sara Domínguez-Rodríguez, PhD, Biostatistician Instituto de Investigación Sanitaria Hospital 12 de Octubre (imas12), Fundación Biomedica del Hospital Universitario 12 de Octubre (FBHU12O). Sección de Enfermedades Infecciosas Pediátricas. Servicio de Pediatría, Hospital Universitario 12 de Octubre. Unidad Pediátrica de Investigación y Ensayos Clínicos (UPIC). Lilit Manukyan, PhD Project Manager. Servicio Madrileño de Salud (SERMAS), Instituto de Investigación Sanitaria Hospital 12 de Octubre (imas12), Fundación Biomedica del Hospital Universitario 12 de Octubre (FIB-H12O). Sección de Enfermedades Infecciosas Pediátricas. Servicio de Pediatría, Hospital Universitario 12 de Octubre. Unidad Pediátrica de Investigación y Ensayos Clínicos (UPIC). Scientific Advisor, Luis Manuel Prieto Tato, MD, PhD, Pediatrician, Associate Professor. Servicio Madrileño de Salud (SERMAS), Instituto de Investigación Sanitaria Hospital 12 de Octubre (imas12), Fundación Biomedica del Hospital Universitario 12 de Octubre (FBHU12O). Sección de Enfermedades Infecciosas Pediátricas. Servicio de Pediatría, Hospital Universitario 12 de Octubre. Unidad Pediátrica de Investigación y Ensayos Clínicos (UPIC). Trial Pharmacologist, Aranzazu Sancho López, MD, PhD,Clinical Pharmacologist Pharmacology Unit. Hospital Puerta de Hierro. Madrid, Spain. Servicio Madrileño de Salud Olivier Marcy. Université de Bordeaux (UoB). France. Valeriane Leroy. Institut National de la Santé et de la Recherche Médicale (INSERM). CERPOP, Toulouse, France. Alessandra Nardone. Penta Foundation (PENTA). Italy. David Burger. Radboudumc University (RUMC). The Netherlands. Quique Bassat. ISGlobal, Hospital Clínic - Universitat de Barcelona, Barcelona, Spain. Centro de Investigação em Saúde de Manhiça (CISM), Maputo, Mozambique ICREA, Pg. Lluís Companys 23, 08010 Barcelona, Spain. Pediatrics Department, Hospital Sant Joan de Déu, Universitat de Barcelona, Esplugues, Barcelona, Spain Consorcio de Investigación Biomédica en Red de Epidemiología y Salud Pública (CIBERESP), Madrid, Spain Matthew Bates. University of Lincoln. United Kingdom. Raoul Moh. Programme PAC-CI. Ivory Coast. Pui-Ying Iroh Tam, DMed, FAAP, FPIDS, FIDSA. Malawi-Liverpool Wellcome Programme (MLW). Liverpool School of Tropical Medicine (LSTM). Kamuzu University of Health Sciences (KUHeS). Malawi. irohtam@mlw.mw Tisungane Mvalo, MBBS, MMED, FC Paed. Lilongwe Medical Relief Trust (LMRF, UNC Project Malawi, Lilongwe. Malawi. Justina Magallhaes. Centro de Investigação em Saúde da Manhiça/Fundação Manhiça (CISM-FM). Mozambique. W. Chris Buck, MD, MSPH. Associate Professor, University of California Los Angeles David Geffen School of Medicine. Universidade Eduardo Mondlane (UEM). Mozambique. wbuck@mednet.ucla.edu Jahit Sacarlal, MD, PhD. Department of Microbiology, Faculty of Medicine, Universidade Eduardo Mondlane (UEM), Maputo, Mozambique. jahityash2002@gmail.com Victor Musiime. MBChB, MMed, PhD Associate Professor, Makerere University, College of Health Sciences, Department of Paediatrics and Child Health,Uganda. Senior Research Consultant, Joint Clinical Research Centre, Uganda. musiimev@yahoo.co.uk Chishala Chabala. MBChB, MSc, MMed. School of Medicine, University of Zambia, Lusaka. Zambia. Hilda Angela Mujuru. University of Zimbabwe. Zimbabwe.
Name and contact information for the trial sponsor {5b}	Servicio Madrileño de Salud (SERMAS)-Fundación para la Investigación Biomédica Hospital Universitario 12 de Octubre (FI+12). Av. de Córdoba, s/n, 28041 Madrid. Spain.
Role of sponsor {5c}	Sponsor Servicio Madrileño de Salud (SERMAS)-Fundación para la Investigación Biomédica Hospital Universitario 12 de Octubre (FI+12) is assuming overall responsibility for the initiation and management of the trial. Funder The funder will have no role in the trial design, conduct, data analysis and interpretation, manuscript writing, and dissemination of results. EDCTP expects that grant holders will disclose the summary results of the study within 12 months from primary study completion (the last visit of the last subject for the collection of data on the primary outcome).

## Introduction

### Background and rationale {6a}

#### Background

According WHO Global Health Observatory Data Repository, pneumonia remains the main cause of death in children in the post-neonatal period, with nearly 800,000 premature deaths globally, each year. According to UNAIDS, the global number of deaths is 110,000 to 120,000 per year including 40,000 deaths due to tuberculosis (TB), despite prompt antiretroviral treatment (ART) initiation. In Africa, 10% of infants die during the first year of life even if on early ART [1, 2]. Pneumonia is the primary cause of death in these children [3, 4].

Currently, the World Health Organization (WHO) guidelines recommend treating severe pneumonia in HIV-infected infants with empirical treatment against *Pneumocystis jirovecii*, *S. pneumoniae*, and *Haemophilus influenzae b* [5]. This approach has decreased mortality in this group of children. However, acute mortality remains unacceptably high, reaching 35% [6].

Cytomegalovirus (CMV) and TB are other important causes of death, still heavily under-recognized, and as such undertreated in this population [7]. CMV has been described as the second most important cause of death among HIV-infected infants with pneumonia under 6 months of age, accounting for around 10–30% of all deaths [7, 8]. Untreated, CMV pneumonia has a mortality of up to 88% [8]. Although the benefit of CMV-antivirals for patients with presumed CMV pneumonia has been proposed to reduce mortality up to 50% in observational studies and trials in immunocompromised patients, there are not, to our knowledge, clinical trials testing this hypothesis in HIV-infected children or adults [6, 8–10]. In adults with HIV infection and CMV viremia, the use of pre-emptive anti-CMV therapy could improve end-organ disease or death [10].

Prevalence of TB in acute pneumonia is around 15%, and at least 75% of them are unrecognized TB [11–13], being the mortality of this group over 80% [14].

In summary, CMV and TB remain significant, but hidden and undertreated, killers of HIV-infected children presenting with pneumonia.

The clinical trial presented here focuses on the search for new therapeutic approaches for the treatment of severe pneumonia in HIV-infected infants.

#### Rationale

CMV disease was the second most common cause of pneumonia death in HIV-infected infants under 6 months of age [5, 7, 8]. However, this pathogen has been so far neglected under the unconfirmed assumption that increasing ART coverage will decrease its prevalence and impact, and due to the current difficulties to confirm its diagnosis. Empirical treatment of CMV pneumonia with valganciclovir appears to be a reasonable option in high-risk populations, as the selected population in this trial. For WHO, improving access to oral systemic treatment with valganciclovir is an explicit priority in children, according to the 2017 Advanced HIV disease Guidelines [15]. Clinical trials aimed at testing this hypothesis are therefore warranted.

On the other hand, HIV-infected infants are a population with a high risk of death due to TB. They have a 23-fold increased risk of pulmonary TB compared to uninfected children, and 10 to 40% of HIV-infected children are estimated to present a TB event during the first year of life in East Africa [16, 17].

According to recent data in children less than 5 years, TB accounts for at least 15% of acute pneumonia [16], but only 30% are confirmed TB. Mortality among the unconfirmed TB group (70%) is 87% when TB is not treated, vs. 6% when treated. Therefore, at least 56% (80% out of 70%) deaths due to TB are avoidable if all patients with TB were treated. Meaning, 8% of acute pneumonia deaths are preventable with empirical TB-T.

In addition, infants have a higher risk of disease progression and death than older children (case fatality ratio of 70% vs. 36%) [18]. The percentage of deaths attributable to TB among HIV-infected children varies from 4.5 to 18% (6, 15, 35-37) [7, 11, 12, 19, 20].

However, these figures are not accurate for infants as autopsy studies seldom have information from younger children. As a result, we know that TB is an important cause of death, but the true impact remains unknown and probably heavily underestimated in this population. The current standard of care (SoC) includes microbiological testing for TB in children with suspected TB; however, with this approach, a significant number of TB remains undiagnosed mainly in infants. Related research is ongoing to evaluate the use of a systematic and rapid TB diagnosis with Xpert MTB/RIF (TB-Speed Project, funded by UNITAID) in children with severe pneumonia. Given the challenge in diagnosing TB in children, even with molecular techniques and the high associated mortality, a new approach including empirical treatment may be lifesaving among HIV-infected infants. Systematic empirical treatment against TB in severely immunosuppressed HIV-infected patients without evidence of active TB disease is an open question that is being assessed in a randomized trial in adults, but currently, there are no similar activities addressed to infants [21]. Even considering the potential downside of empirical treatment (resistance development, potential toxicity), empirical treatment against CMV and TB seems worthy to be investigated.

Also, some recent research suggests that CMV accelerates disease caused both by HIV and TB. Plausibly, this deleterious interaction can be stopped with treatment against CMV. CMV and TB may also co-infect the same child, and the impact of these co-infections is poorly understood. Several studies point out that the immune response elicited by CMV may be associated with the risk of developing TB and TB disease progression [22–25].

### Objectives {7}

#### Hypothesis

Empirical treatment against CMV with oral valganciclovir and empirical treatment against TB (TB-T) together with standard pneumonia treatment improve survival in HIV-infected infants with severe pneumonia, with low risk/benefit balance.

#### Primary objective

The primary objective is to compare the impact on 15-day and 1-year mortality of combined systematic empirical treatment against TB and CMV plus SoC versus SoC in HIV-infected infants with severe pneumonia.

#### Secondary objectives

Secondary objectives are as follows:

##### Clinical

- To compare the impact on 15-day and 1-year mortality of systematic empirical valganciclovir plus SoC versus SoC in HIV-infected infants with severe pneumonia.
- To compare the impact on 15-day and 1-year mortality of systematic empirical TB-T plus SoC versus SoC in HIV-infected infants with severe pneumonia.
- To compare the cumulative days of oxygen therapy from randomization until discharge of the intervention arms versus SoC.
- To compare the cumulative number of days of hospitalization 1 year after randomization of the intervention arms versus SoC.

##### Pharmacovigilance

- To evaluate the safety of empirical valganciclovir and empirical TB-T in HIV-infected infants hospitalized with severe pneumonia of the intervention arms versus SoC.

##### Epidemiological

- To know the prevalence of CMV infection in HIV-infected infants hospitalized with severe pneumonia.
- To know the prevalence of confirmed and unconfirmed TB in HIV-infected infants hospitalized with severe pneumonia.
- To know the incidence of confirmed and unconfirmed TB in HIV-infected children hospitalized with severe pneumonia during a 1-year follow-up.
- To know the prevalence of CMV infection and confirmed and unconfirmed TB in HIV-infected children hospitalized with severe pneumonia that died.

##### Molecular response to treatment

- To assess the decrease of the quantitative CMV viral load in blood and saliva in HIV-infected infants hospitalized with severe pneumonia treated with valganciclovir.

##### TB diagnosis

- To assess the diagnostic accuracy of TB-LAM for the diagnosis of confirmed TB (reference: positive Xpert MTB/RIF Ultra in feces and/or nasopharyngeal aspirate (NPA)).

##### Economic evaluation

- To analyze the cost-effectiveness of the proposed treatment strategies in each context.

### Trial design {8}

This is a Phase II-III, open-label randomized factorial (2 × 2) clinical trial, to be conducted in six countries, namely Ivory Coast, Malawi, Mozambique, Uganda, Zambia, and Zimbabwe, in collaboration with research organizations from Spain, France, UK, Italy, and The Netherlands. The study aims to enroll 624 HIV-infected infants altogether.

A factorial clinical trial is being proposed for this clinical trial. This design provides the advantage of assessing the independent and cumulative effect of the interventions. Each intervention targets a different mechanism of impact on the primary endpoint. Therefore, rather than the common assumption of no interaction between the two interventions in factorial trials, we do anticipate a better performance of the combined arm.

The study will be a classically defined as phase II–III as:

The empirical treatment against CMV (valganciclovir) is a phase II, according to Food and Drug Administration (FDA) definitions, as the purpose is to investigate efficacy and side effects in up to several hundred people with the disease/condition (presumed CMV pneumonia).

The empirical treatment against TB (isoniazid, rifampicin, pyrazinamide, and ethambutol) is a confirmatory phase III since the trial is aimed to demonstrate whether a product offers a treatment benefit to a specific population (in this case, HIV-infected infants with unknown-etiology severe pneumonia).

**Fig. 1:**
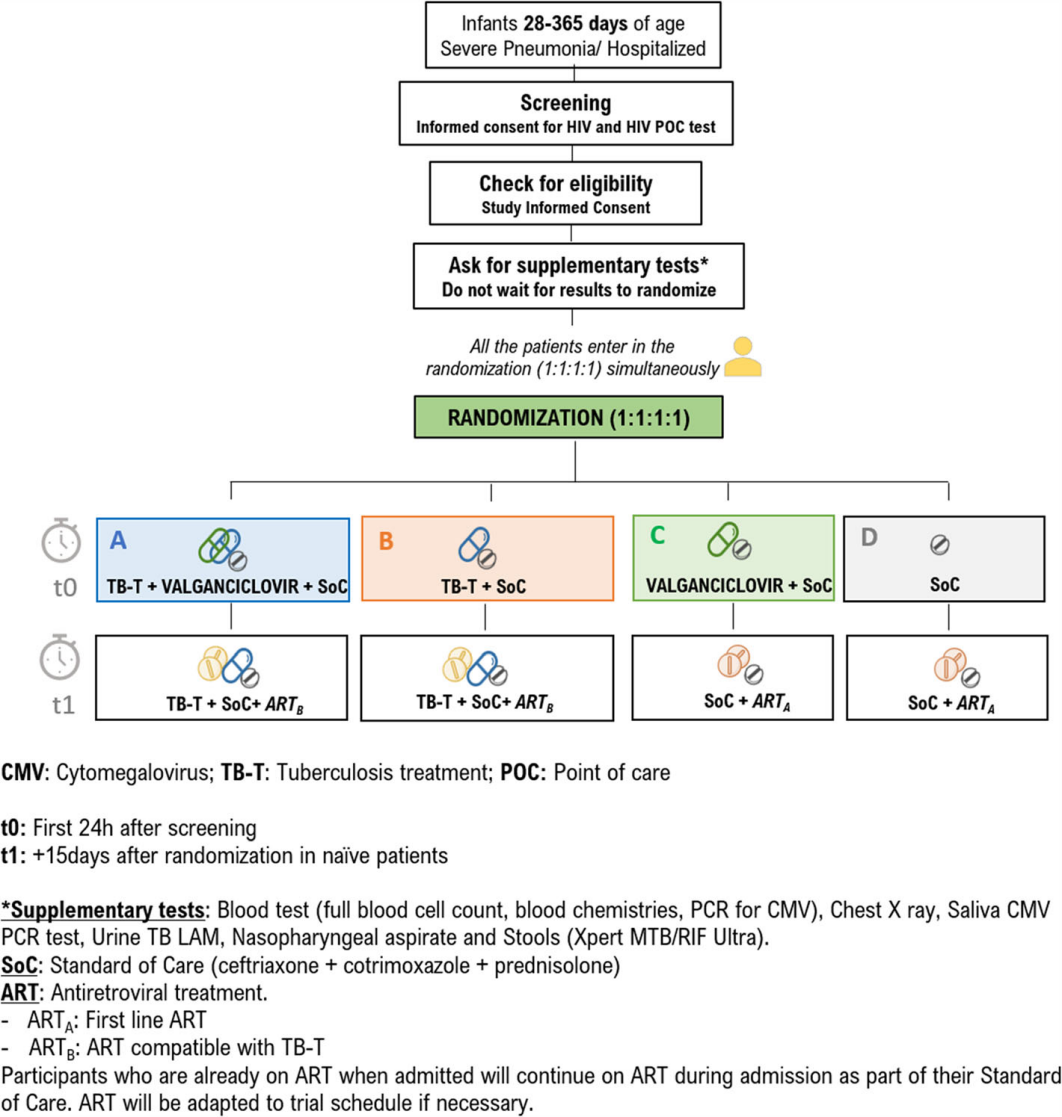
Trial flowchart

Number of arms (See Fig. 1, Trial flowchart): The trial will have four arms: randomization 1:1:1:1. Participants will be randomized simultaneously to receive or not receive each of the two interventions:

- Treatment against CMV: valganciclovir plus SoC, or SoC for 15 days. Open.
- Treatment against TB: TB-T plus SoC, or SoC for 6 months. Open.

In the case of a patient randomized to SoC or valganciclovir, if any information arrives after randomization suggesting or confirming TB, this patient can initiate TB-T and will remain in the assigned arm.

## Methods: participants, interventions, and outcomes

### Study setting {9}

EMPIRICAL will be mainly performed in East and Southern Africa, the region that is hardest hit by HIV and TB. To cover other population a country from West Africa (Ivory Coast) has also been included. According to UNICEF, there are an estimated 2.1 million HIV-infected children worldwide, more than 80% of whom live in sub-Saharan Africa. Despite significant progress, due to the prevention of mother-to-child transmission (PMTCT) programs (estimated to have reduced by 66% reduction of perinatal HIV in this area), the number of children becoming newly infected with HIV remains unacceptably high (around 150,000) in sub-Saharan Africa [26].

The trial sites have been selected to provide a high number of infants with HIV infection with severe pneumonia in regions where TB rates are extremely high. Moreover, the selected sites have conducted previous studies of high public health relevance in the African continent: Mozambique, Malawi, Zambia, Zimbabwe, Uganda, and Ivory Coast.

Partners include seven academic institutions associated with 21 enrolling secondary and tertiary hospitals:

1. Universidade Eduardo Mondlane. Enrolling sites: Hospital Central de Maputo, Hospital Geral José Macamo, Hospital Provincial da Matola, Hospital Geral de Mavalane, Nampula Hospital Central Beira. Tertiary care hospitals.
2. Centro de Investigação em Saúde de Manhica (CISM). Enrolling site: Manhiça District Hospital (MDH), Xai Xai Hospital. Secondary care hospitals.
3. Liverpool school of tropical medicine (LSTM) (Malawi-Liverpool Wellcome Trust Clinical Research Program) Enrolling site: Queen Elizabeth Central Hospital. Tertiary Hospital. The following referral hospitals will be included in the trial: Hospitals: Chikwawa District Hospital, Thyolo District Hospital and Chiradzulu District Hospital.
4. Lilongwe Medical Relief Trust. Enrolling site: Kamuzu Central Hospital (KCH).
5. HerpeZ Zambia. Enrolling sites: University Teaching Hospital (UTH), and Arthur Davidson Children Hospital. Tertiary care hospitals.
6. University of Zimbabwe Clinical Research Centre (UZ-CRC), Enrolling sites:

Harare Central Hospital, Parirenyatwa Group of Hospitals pediatric wards. Tertiary care Hospitals.

(7) Makerere University. Enrolling sites: China Uganda Friendship Hospital Naguru, Jinja Regional Referral Hospital, and Mbarara Regional Referral Hospital. Tertiary Hospitals.
(8) PACCI Programme. Enrolling sites: Cocody University Hospital Center (CHU), Treichville CHU and Angré CHU. Tertiary Hospitals.

### Eligibility criteria {10}

#### Screening criteria

HIV-infected infants from 28 to 365 days of age, admitted with pneumonia.

#### Inclusion criteria

Must fulfill all five (5):

1. Age 28 days to 365 days of age
2. Pneumonia defined as chest indrawing or fast breathing for age, for infants 28 to 60 days of age ≥ 60 breaths per minute and for infants 61 to 365 days of age, ≥50 breaths per minute.
3. Current hospitalization with pneumonia with criteria for parenteral antibiotics (1 or more criteria)

- Chest indrawing with HIV infection
- No improvement with oral treatment.
- One or more danger signs according to WHO [5, 27, 28]
  Central cyanosis or saturation of O_2_ < 90%
  Severe respiratory distress, e.g., grunting or very severe chest indrawing
  Signs of pneumonia with a general danger sign:
  Unable to drink or breastfeed
  Persisting vomiting
  Convulsions in the last 24 h
  Lethargic or unconscious
  Stridor while calm
  Severe malnutrition

(4) HIV-confirmed infection (with at least one molecular method: DNA PCR or RNA PCR/viral load).
(5) Informed consent obtained

#### Exclusion criteria

- Clinical TB (pulmonary or extrapulmonary) diagnosis, defined as the necessity of TB-T prescribed by a physician, at the moment of randomization
- Known bacteriologically confirmed TB case (at least one biological specimen positive by culture or Xpert MTB/RIF) at the moment of randomization.
- Patient previously treated for TB or currently on treatment for TB.
- Documented evidence of close TB exposure (household contact of a patient with documented TB during the lifetime of the child, or currently receiving TB-T).
- Pure wheezers defined as a clear clinical improvement after a bronchodilator test (give a challenge of rapid-acting inhaled bronchodilator for up to three times 15–20 min apart. Count the breaths and look for chest indrawing again, and then re-classify).
- Active malignancies.
- Systemic immunosuppressive medications. Steroids will be considered to be immunosuppressing only if > 2 mg/kg of prednisone or equivalent during > 15 days.
- Evidence of condition other than HIV and pneumonia which precludes, to the judgment of the clinical researcher, enrollment in this trial due to risk for the patient. In case of doubt, the Trial Management Team will be contacted to assess eligibility.
- Less than 2.5 kg of weight.
- Hemoglobin < 6 g/dL in the screening blood test or in a test done in the last 48 h. Transfusion is permitted to achieve > 6 g/dL if the patient’s state allows it. In case a transfusion is administered, the patient can be enrolled.
- Neutropenia < 500/mm [3] in the screening blood test or in a test done in the last 48 h. Repeating the test is allowed to check eligibility.

Patients should be enrolled as soon as possible after admission; however, it is admissible to include patients at any time during admission as long as they fully filled the inclusion criteria and none of the exclusion criteria at the time of the recruitment. Patients that are referred from other centers can be recruited. Children already on ART can be enrolled.

### Who will take informed consent? {26a}

Appointed members of each local research team who have received appropriate training will obtain written informed consent from the caregiver of the patient before being enrolled in the study.

For the purposes of this protocol, “caregiver” refers to the legally authorized representative (LAR) of the child and informed consent may only be obtained from a child’s LAR. Both mother and father are considered LARs for a child so that consent may be obtained from either parent. In the absence of a biological parent, all efforts will be made to get documented proof of legal guardianship that would be needed to establish a caregiver’s status as a LAR. In the case of parents who were younger than the legal age for signing, one of the parent’s LAR will be asked for consent and the assent will be asked to the mother or father of the child. This study will have one main informed consent form (ICF) for participation in the study and one ICF for screening procedures. Specific ICF for screening procedures will be needed only at some sites, if at the time of recruitment the child does not have data of his/her HIV status, hemoglobin level, and neutrophil count in those sites where the screening study procedures are not included in the standard of care. In case the clinician asks for these tests outside the standard of diagnosis of the center, the screening consent will be asked.

Caregivers of HIV-infected infants will be given an information sheet about the EMPIRICAL trial and asked to give written consent before any trial-specific procedures are performed or any sample is taken for the trial.

Where the LAR cannot read or write or require translators, appropriate alternative methods for supporting the informed consent process will be employed to ensure that caregivers of children fully understand what will and may happen to their child while participating in a research study. It may include allowing a witness to sign on a participant’s behalf (in the case of inability of reading or writing), and/or providing Participant Information Sheets in local languages.

If any new information is learned that may affect the caregiver’s decision to stay in the trial this information will be shared with the caregivers in writing.

All consent materials will be approved by the appropriate correspondents’ Ethics Committees before any use.

### Additional consent provisions for collection and use of participant data and biological specimens {26b}

Other sub-studies and ancillary studies will be performed in the frame of the study in some of the sites upon condition that the specific consent has been provided by the LAR for any of the sub-studies.

Participants will be asked to consent to the given options concerning their participation in the study including participating in the main trial but not be involved in any of the sub-/ancillary studies or participate in one or a few of them.

Caregivers of dead participants eligible for participation in the Cause of Death minimally invasive autopsy sub-study will be provided the specific informed consent only in case of death. Information about the need for sending samples to Microbiological and Pathology research laboratories associated with ISGlobal in Barcelona (Spain) will be included.

Caregivers of eligible infants for participation in PK sub-studies will be provided the specific informed consents. Information about the need for sending samples to the Department of Pharmacy of Radboud University Medical Center, Nijmegen (Netherlands) will be included.

## Interventions

### Explanation for the choice of comparators {6b}

Valganciclovir is the only oral available agent for the treatment of CMV. Valganciclovir has been used with success in young infants for congenital CMV in several trials, is often used off-label, and now is widely used in this subset of patients for congenital CMV. Valganciclovir is also used off-label for the treatment of CMV pneumonia.

#### Valganciclovir

Valganciclovir hydrochloride 50 mg/ml, powder for oral solution, is approved by European Medicines Agency (EMA) for the treatment of CMV retinitis in adult patients with AIDS and the treatment and prevention of CMV disease in seronegative adults and children recipients of a seropositive CMV solid organ transplant. Valganciclovir has a Pediatric Investigation Plan completed by 2013 (EMEA-000726-PIP01-09-M02, compliance checked on 10/11/2013). It is also approved by the FDA for the treatment of adults with CMV retinitis in patients with AIDS and the prevention of CMV disease in kidney, heart, or kidney-pancreas transplant adult patients at high risk. For children, it is authorized for the prevention of CMV disease in kidney or heart transplant patients at high risk. Currently, valganciclovir is not authorized by any Regulatory Agency for the (empirical) treatment of suspected CMV pneumonia in children with HIV.

Valganciclovir for CMV retinitis is included in the WHO essential medicines list [29].

#### Isoniazid, rifampicin, pyrazinamide, and ethambutol

This combination of drugs is the first choice for the treatment of tuberculosis in children. All isoniazid, rifampicin, pyrazinamide, and ethambutol are currently authorized as a single dose or FDC across all relevant regulatory agencies in the world for the treatment of TB infection in adults and children, including infants.

### Intervention description {11a}

Standard of care (SoC) will always include antiretroviral treatment (ART), antimicrobial therapy, and treatment for *P. jirovecii* pneumonia, and TB-T if any information arrives after randomization suggesting or confirming TB.

Once patient confirmation of eligibility and the criteria for randomization have been met, patients will be centrally randomly allocated in a 1:1:1:1 fashion to one of four possible treatment combinations, i.e.,

- SoC + TB-T + valganciclovir
- SoC + TB-T
- SoC + valganciclovir
- SoC

The researcher nurse/assigned staff will receive and administer the drug/s according to prescription. Medications derived from randomization will need to be administered within the first 24 h after randomization.

#### IMPs

(A) Valganciclovir hydrochloride (Roche^TM^) 50 mg/mL, powder for solution
(B) TB-T:
  Induction phase (2 months): Fixed-dose dispersible tablet of rifampicin, isoniazid, pyrazinamide (75/50/150 mg) (Macleods^TM^) and Ethambutol 100 mg tablet (Macleods^TM^)
  Continuation phase (4 months): Fixed-dose dispersible tablet of rifampicin/isoniazid (75/50 mg) (Macleods^TM^)

Appropriate dosages will be determined individually, based on the recommended dosing in the Summary of Product Characteristics (SmPC) for each investigational TB study drug.

For valganciclovir, the recommended dosing for infants in the treatment of CMV has been established based on available literature [30].

#### Doses

(A) Valganciclovir (powder for suspension, 50 mg/mL) oral, 16 mg/kg/12 h for 15 days, orally or via nasogastric or orogastric tube.
(B) Oral anti-tuberculosis drugs standard doses are:

- Isoniazid 10 mg/kg (range 7–15 mg/kg)/day; maximum dose 300 mg/day for 6 months.
- Rifampicin 15 mg/kg (range 10–20 mg/kg)/day; maximum dose 600 mg/day for 6 months.
- Pyrazinamide 35 mg/kg (range 30–40 mg/kg)/day for 2 months.
- Ethambutol 20 mg/kg (range 15–25 mg/kg)/day for 2 months

TB-T will be administered orally or via nasogastric or orogastric tube following doses shown in Table 1 once per day.

**Table 1:** Dosing table for TB drug tablets

Weight band^a^	Numbers of tablets		
	Intensive phase RHZ 75/50/150	Intensive phase Ethambutol 100^b^	Continuation phase RH 75/50
4–7 kg	1	1	1
8–11 kg	2	2	2
12–15 kg	3	3	3
16–24 kg	4	4	4
25+ kg	Adult dosages recommended		

^a^ Infants below 4 kg will receive half FDC and half ethambutol pill. The safety and efficacy of this formulation have not been assessed in large studies in infants, but equivalent liquid formulations are used routinely. A PK sub-study (PK 4) to investigate the relationships between age, peak concentrations of TB agents, and efficacy/toxicity to guide the treatment with FDC in infants weighing lower than 4 kg will be done. As soon as a result arises, the dosage will be updated if necessary

^b^Ethambutol should be added in the intensive phase for all recruited children following WHO recommendation as they are HIV-infected

For adherence, note that completing 80% of all scheduled dose administrations is considered to meet treatment completion criteria (i.e., 8 out of 10 doses over the 5 days). If doses are missed due to non-adherence, the dose will be recovered beyond documenting the missed doses and counseling the caregiver on adherence and study product administration.

### Criteria for discontinuing or modifying allocated interventions {11b}

#### Anemia

Valganciclovir will be withheld at any moment if the hemoglobin count reproducibly decreases to < 6 g/dl. After the hemoglobin count increases to ≥6 g/dl, valganciclovir administration at the standard dose may resume.

#### Neutropenia

If absolute neutrophils count reproducibly decreases to ≤500 cells/mm^3^ valganciclovir will be withheld until the neutrophils recover to > 750 cells/mm^3^, and then the administration of the drug will resume at the standard dose.

#### Thrombocytopenia

Valganciclovir will be withheld if the platelet count reproducibly decreases to ≤50,000/mm^3^. After the platelet count increases to ≥50,000/mm^3^, valganciclovir administration at the standard dose may be resumed.

#### Hepatotoxicity

If ALT levels are ≥5 times the upper limit of normal (with or without symptoms) or ≥ 3 times normal in the presence of symptoms, (including nausea, vomiting, right upper quadrant pain or lethargy) all TB-T will be stopped immediately, and the patient will be evaluated carefully according to local practices. The treatment will be considered for reinitiating in a stepwise way.

In case of significant drug-induced liver injury, cotrimoxazole and other potential hepatotoxic medication, including valganciclovir, will also be stopped. However, rechallenge with the same anti-TB medications is not recommended for those who have had fulminant hepatitis (defined as hepatic encephalopathy with coagulopathy). Rechallenge of ART and other potential hepatotoxic medication will be considered. Management of each severe case will be individualized and discussed with the central Clinical Trial Unit and Data Safety Monitoring Board (DSMB) if necessary.

No modifications of dosage are allowed unless very justified and previous consultation with the CTU A dose modification will result in the potential exclusion of the participant in the per treatment analysis if received < 50% of the expected dose.

#### Renal impairment

If renal function is normal, then administer the full dose of valganciclovir at the same intervals (16 mg/kg/dose BID). If renal function is moderately impaired, then administer the full dose of valganciclovir at decreased intervals (16 mg/kg/dose administered once daily). If renal function is severely impaired, then discontinue study medication.

#### Cutaneous drug reactions

If patients develop drug rash on the trial, this could be potentially related to cotrimoxazole (a frequent cause of drug rashes), valganciclovir, ART, or one of the TB drugs. For very mild rashes they will be monitored with regular review and symptomatic therapy. In patients with more significant symptoms at presentation or who develop more severe clinical features (extensive rash, fever, systemic symptoms, blistering or desquamation, angioedema or mucosal involvement), all potential culprit drugs will be stopped immediately. Patients will be managed according to local practices and in consultation with the CTU and DSMB if necessary. Re-challenge of TB drugs after a severe drug rash will be discussed with the CTU and DSMB if necessary.

#### Known drug reactions and interaction with other therapies

Since the IMP and some of those that could be used as concomitant SoC medication are metabolized in the liver by CYP enzymes, there is a risk for drug-drug interactions. Therefore, investigators will be encouraged to follow clinical practice guidelines and SmPC recommendations when choosing between the ART options available. Close monitoring of potential risks derived from anticipated drug reactions or interactions will be conducted. A specific pharmacovigilance plan will be performed.

### Strategies to improve adherence to interventions {11c}

Strategies to improve adherence to the intervention protocol will be implemented. Face-to-face adherence reminder sessions will take place at the product dispensing in each visit. Instructions will be given about taking study pills, timing, storage, importance, what to do in the event of a missed dose, notification of pill counts at visits, and calling the clinic if problems with study medications. Discussion of reasons for missed doses, adherence, and linking pill to daily activities will be held.

Adherence assessments will be done at every visit including pharmacy files and questionnaires.

For caregivers for whom adherence is an issue, individual adherence counseling will be given to optimizing therapy. If doses are missed due to non-adherence, the documented missed doses will be recovered (in case of TB-T) and counseling the caregiver on adherence and study product administration will be done.

If the patient vomits after taking the study medication in the following ½ hour, the administration can be repeated twice. If a child vomits after three attempts, that child will be referred to care for a work-up of the vomiting cause.

Following hospital discharge, caregivers will be asked structured questions to assess ART and TB-T adherence. Adherence to any medication will be measured as doses missed or vomited/spitted in the first 30 min after administration, without replacement.

### Relevant concomitant care permitted or prohibited during the trial {11d}

#### Concomitant therapy (SoC package)

##### Treatment of WHO-defined clinical severe pneumonia

- Ceftriaxone is recommended as a first-line antibiotic regimen for HIV-infected infants with chest indrawing pneumonia or severe pneumonia and will be used in the clinical trial as SoC bacterial pneumonia treatment. Duration of treatment will be at least 5 days according to WHO recommendations [5].
- Alternatively, ampicillin (or penicillin when ampicillin is not available) plus gentamicin could be used. For HIV-infected with chest indrawing pneumonia or severe pneumonia, who do not respond to treatment with ampicillin or penicillin plus gentamicin, ceftriaxone alone is recommended for use as second-line treatment. Duration of treatment will be at least 5 days according to WHO recommendations.
- Switch to the oral antibiotic will be to amoxicillin 80 mg/kg/day twice a day as per WHO recommendations. The switch to oral antibiotic should be evaluated when the infant:
  Has become hemodynamically stable
  Has clinically improved
  Is able to ingest medications
  Has acceptable functioning of the gastrointestinal tract

Therefore, all patients will receive immediate enhanced pneumonia treatment which is considered the SoC among HIV-infected infants:

- First choice: ceftriaxone 80 mg/k/day iv or im (if parenteral access has not been secured) for at least 5 days.
- Second choice, only if ceftriaxone is not available: ampicillin 50 mg/kg, or benzylpenicillin 50,000 unit/kg im/iv every 6 h plus gentamicin 7.5 mg/kg/im or iv once a day, at least 5 days.

##### *P. jirovecii* pneumonia (PCP) empirical treatment

Empiric cotrimoxazole treatment for PCP is recommended as an additional treatment for HIV-infected infants aged less than 1 year with chest indrawing and severe pneumonia. Therefore, cotrimoxazole po/iv (trimethoprim (TMP) 8 mg/kg/dose + sulfamethoxazole (SMX) 40 mg/kg/dose three times daily for 21 days) will be added as the SoC treatment.

A recent clinical trial from Malawi showed a strong benefit associated with the use of corticosteroids together with cotrimoxazole for PCP treatment [31]. In most of the study sites (6/8), steroids are already used for PCP treatment and are considered SoC. Therefore, oral prednisolone (2 mg/kg for 7 days, plus 1 mg/kg other 7 days, plus 0.5 mg/kg for 7 days for a total of 21 days) or equivalent will be added to the SoC treatment of the study in all the sites.

After finishing the recommend treatment against PCP prophylaxis with cotrimoxazole (TMP 4 mg/kg/day + SMX 30 mg/kg/day) will be established following the WHO guidelines [32].

##### Antiretroviral treatment for HIV infection

If the child is naïve, or not taking prescribed ART, ART will be started in all HIV-infected infants according to the WHO and national guidelines on day 15 ± 7. ART regimens will be based on what is being used in national programs, as this will ensure that participants can stay on the same regimens. The recommended regimen for naïve patients is ABC + 3TC + LPV/RTV. Patients already on ART are eligible for recruitment into the study.

Due to the interactions between rifampicin and LPV/RTV, infants co-infected with HIV and TB, or those randomized to receive empiric TB treatment must receive the best available therapy, including preferably one of these possibilities (all supported by the WHO recommendations) [32, 33]

- AZT (or ABC) + 3TC + Dolutegravir
- ABC + 3TC + super-boosted LPV/RTV (1:1)
- ZDV + 3TC + super-boosted LPV/RTV (1:1)
- Triple NRTI (ZDV + 3TC + ABC)

Regimens discouraged, although permitted if there is no better option at the moment and are allowed by the national guidelines, include:

- ABC + 3TC + NVP full dose
- AZT + 3TC + NVP full dose
- AZT (or ABC) + 3TC + double dose LPV/RTV (4:1).

There is ongoing research into the efficacy of a 2 NRTI plus LPV/RTV (4:1) dosed every 8 h during rifampicin-based TB treatment. ART regimens consistent with the local guidelines will be initiated with appropriate adaptation for concurrent TB medication and other treatments. Study staff will oversee the prescription of the ART regimen. In the instance of a local drug shortage or previous ART prescription, the study staff together with clinical staff will decide the therapeutic options for potential drug substitutions, based on current medical literature. The safety coordinator of CTU will be available to providing advice if needed.

ART will be prescribed by study staff together with clinical staff 15 ± 7 after enrollment and will be administered by caregivers after discharge or by hospital staff during hospitalization.

##### Isoniazid preventive therapy (IPT)

Since 2011, WHO has issued recommendations regarding IPT in HIV-infected children. Children older than 12 months and unlikely to have TB disease on symptom-based screening and no contact with a TB case must receive 6 months of IPT if they are living in a high TB prevalence setting (*strong recommendation, low-quality evidence*). In addition, children younger than 12 months who have contact with a TB case and where TB disease has been ruled out (using investigations) should receive 6 months of IPT (*strong recommendation, low-quality evidence*). If this situation is known at the time of screening, these children are not available for inclusion in the study.

These recommendations have higher evidence in children without ART [34]. However, the evidence of benefit is weak for children on ART [35]. Existing challenges in ruling out TB, concerns that IPT may promote isoniazid resistance, and economic and health system constraints have contributed to hurdles in the implementation of IPT among children HIV-infected and on ART. Therefore, even in countries with high TB incidence, IPT is often not provided. For the patients randomized to no TB-T who become older than 12 months during the follow-up of the trial, the policy of this trial is to keep current local practice, which is not to routinely administer IPT in most of the sites. The centers that have already the recommendation in place can provide or not IPT at the discretion of the attending researcher/physician. Also, patients younger than 12 months who turn to have household contact with confirmed TB during follow-up will have to be evaluated for TB. If the evaluation shows no TB disease, they should receive IPT, according to local protocols.

If given, isoniazid should be administered at a dose of 10 mg/kg per day, range 7–15 mg/kg, maximum dose 300 mg/day. If available, vitamin B6 should be supplied with isoniazid at a dose of 25 mg daily [35]. A specific analysis will be done to evaluate the influence of isoniazid in patients randomized to “No TB-T” but on isoniazid.

##### CPAP / mechanical ventilation

The use of cannulas with positive-airway pressure (CPAP) or mechanical ventilation will be permitted based on local practices. Information will be collected at the individual level and incorporated into the final analysis, as these systems may influence acute mortality.

### Provisions for post-trial care {30}

The Sponsor holds insurance against claims from participants for injury caused by their participation in the clinical trial. Participants may be able to claim compensation if they can prove that the Sponsor has been negligent. However, as this clinical trial is being carried out in a research facility/clinic, the research facility/clinic continues to have a duty of care to the participant of the clinical trial. The Sponsor does not accept liability for any breach in the research facility/clinic’s duty of care or any negligence on the part of research facility/clinic employees.

Participants may also be able to claim compensation for injury caused by participation in this clinical trial without the need to prove negligence on the part of the Sponsor or another party. Participants who sustain an injury and wish to claim for compensation should do so in writing in the first instance to the Chief Investigator, who will pass the claim to the Sponsor’s Insurers.

After the last visit, families will be referred to the HIV Treatment Program to continue their care.

There is no need for provision of treatment post-trial as the ART is not IMP and is given by the government and the IMP pneumonia treatment will have finished long before the end of the trial. Clinical sites have integrated clinical referral for children between care and research programs to facilitate the transfer of clinical information between the two programs.

### Outcomes {12}

#### Primary endpoint

The primary endpoint of the study is all-cause *mortality*, focusing on the short-term (up to 15 days) and long-term (up to 1 year) mortality. Mortality will be calculated using all-cause mortality after the admission over all the trial time.

Mortality is the most important outcome for patients, their families, and child health programs. Several studies in both adults and children have shown that mortality rates in HIV-infected children are far higher during infancy; if additional interventions can help patients to survive this period, they have a good chance of doing well in the long term.

The format of the outcome will be the mortality rate (density incidence person-year). Mortality will also be described as the proportion of patients who died at 15 days and 1 year after the day of enrollment. We will describe the impact of the treatment on the proportion of children with mortality (beta). Also, proportional time-to-event methods will be explored (hazard ratio).

#### Secondary endpoints/outcomes

For each arm and for each drug, the safety occurrences will be reported following the Safety Definitions of the European Union Directive 2001/20/EC Article 2, based on the principles of ICH-GCP (International Conference on Harmonization of Technical Requirements for Registration of Pharmaceuticals for Human Use-Good Clinical Practice).

1. Clinical
  - Duration of oxygen requirements (in days, from the first requirement until definitive withdrawal, being day 1 the first day of oxygen requirement).
  - Cumulative days of hospitalization from discharge to day + 360 after enrollment
2. Pharmacovigilance
  - The safety endpoints will include:

- Serious adverse events (SAEs) other than death.
- Adverse reactions (AR).
- Adverse events (AEs) requiring stop of an investigational medical product (IMP), all AEs relevant for risk/benefit ratio, including infections, all liver damage, neurological and optic toxicity, renal, hematological, and any AE grade 1, 2, 3, or 4 that the investigator estimates to be relevant.
- Incidence of TB-related immune-reconstitution inflammatory syndrome (IRIS).

(3) Epidemiological
  - Baseline prevalence of CMV infection and CMV-attributable pneumonia (based on a CMV viral load threshold) in recruited HIV-infected infants with severe pneumonia
  - Baseline prevalence of microbiological confirmed and unconfirmed TB (according to Graham criteria, Updated Clinical Case Definitions for Classification of Intrathoracic Tuberculosis in Children 2015) in recruited HIV-infected patients with severe pneumonia (see Additional file 1)
  - New confirmed and unconfirmed TB cases according to Graham criteria during 1 year of follow-up among patients without TB-T
  - Proportion of confirmed and unconfirmed TB, according to Graham criteria, in dead children
  - Proportion of CMV infection in dead children
(4) Molecular response to treatment

- Reduction of quantitative CMV viral load in blood and saliva in infants treated with valganciclovir from enrollment to day + 15

(5) TB diagnosis

- To assess the diagnostic accuracy of TB-LAM for the diagnosis of confirmed TB (reference: positive Xpert MTB/RIF Ultra in feces and/or NPA)

(6) Economic evaluation

- Main outcomes include quality-adjusted life expectancy and per-patient cost

#### Table of endpoints/outcomes

Table 2 shows endpoints/outcomes.

**Table 2:** EMPIRICAL endpoints and outcomes

Variable/outcome	Hypothesis	Outcome measure	Method of analysis
**Primary**	Intervention improves survival at 15d and 1y	All-cause Mortality rate	Generalized Linear Mixed Models
Mortality (rate incidence/person)		Event mortality	Generalized Linear Mixed Models (Logistic)
Mortality (dichotomous)		Time-to-event	Flexible splines survival analysis and Kaplan Meier
**Secondary**			
Serious adverse events	Non-superiority of treatment arms	Event rate (density incidence person-year)	Chi-squared/Fisher-test Generalized Linear Mixed Models (Poisson) Kaplan-Meier
Serious adverse reactions	Non-superiority of treatment arms		
Unexpected Serious Adverse reactions	Non-superiority of treatment arms		
Withdrawal treatment due to AEs	Occurrence	Proportions of withdrawals of treatment	Chi-squared/ Fisher-test
Cumulative days of hospitalization	Improvement with treatment arms	Days of hospitalization	T-test/Kruskal-test
Duration of oxygen requirements	Improvement with treatment arms	Days with oxygen therapy	T-test/Kruskal-test
Incidence of TB-related immune-reconstitution inflammatory syndrome (IRIS)	Occurrence	Proportions of IRIS cases	Chi-squared/ Fisher-test
CMV prevalence	At least 60% of participants	Proportion of participants with PCR+ in saliva	Chi-squared/ Fisher-test
TB prevalence	At least 15% of participants	Proportion of participants with TB test +	Chi-squared/ Fisher-test
TB incidence	Improvement with treatment arms	Proportion of participants with TB	Chi-squared/ Fisher-test
CMV viral load reduction at 15 days	Improvement with treatment arms	Change in qualitative CMV viral load in urine	Chi-squared/ Fisher-test
TB-LAM accuracy	TB-LAM improve TB diagnosis accuracy compare to GeneXpert Ultra in feces and nasopharyngeal aspirate (NPA).	Sensitivity and specificity of both diagnostic techniques	AUC curves and Kappa Index
Death due to TB	Improvement with TB-T	TB death according to the cause of death test	Chi-squared/ Fisher-test
Death due to CMV	Improvement with CMV	CMV death according to the cause of death test	Chi-squared/ Fisher-test
Quality-adjusted life expectancy	Quality-adjusted life expectancy improvement with treatment arms	Quality-adjusted life expectancy	Chi-squared/ Fisher-test
Per-patient cost	Inferiority with treatment arms	Per-patient cost	T-test/Kruskal-test


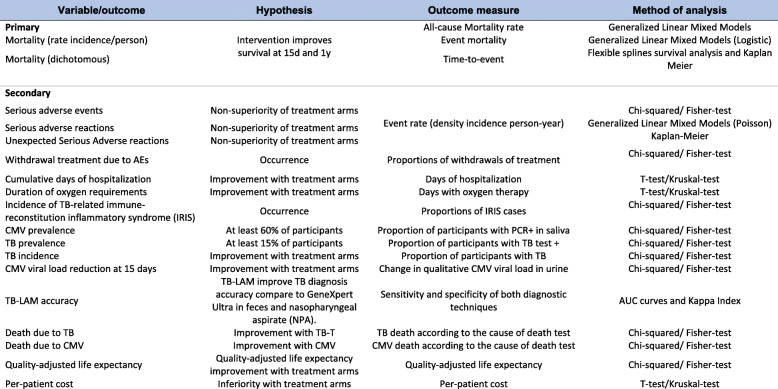


### Participant timeline {13}

The IMP should be started within 24 h after randomization and should be administered as outlined in the following sections of the protocol. The study staff should follow dose modification rules as established in the corresponding SmPC for the management of related or unrelated AEs. All deviations from protocol-specified dose modifications must be documented.

Scheduled visits for data and specimen collection will occur either in the hospital if still in-patient, or at the Outpatient Clinic (for discharged subjects). Following discharge, children will be referred to the pediatric HIV clinic for follow-up, in which a study clinician will be based.

Children will be followed up for a total of 12 months after the day of enrollment. Patients will have follow-up visits at day + 3 after enrollment, at discharge from the enrolling hospital, + 15 days, + 30 days, + 60 days (maintenance phase initiation in TB-T), + 90 days, + 180 days (end of TB-T visit), and + 360 days (“last day, last visit”), unscheduled visits, close-out visit form, and log questionnaires as Treatment log, Adverse event log. If children do not attend the follow-up visits, it means to reach them at the household level or to help them reach the hospital with transport provided by the study staff, as per local practice.

Before discharge, caregivers will be instructed on how and when to administer the study drugs to their child as well as how to contact the study site personnel for concerns that may arise between scheduled visits by phone. Caregivers will receive an instruction sheet with details on the timing and dosing necessary to complete the treatment regimen as well as signs and symptoms that should prompt an immediate call to study staff. A study phone number will be provided to each caregiver.

At each study visit, study staff will administer a standardized questionnaire and will collect relevant clinical and laboratory information from the medical record. Blood specimens will be collected.

Infants will be evaluated at the study facilities during the follow-up visits to assess tolerability and adherence and to monitor AEs. Participants who do not attend any of the scheduled visits will be contacted telephonically by study personnel and through the community to assess the vital status and compliance with study interventions. All efforts will be done to assess the survival of the patients.

After discharge, TB-T will be provided up to the next visit, if randomized to TB-T, and after each visit up to the following visit. All patients will be scheduled equally for follow-up visits. Follow-up will end 12 months after the initial enrollment. Children recruited once will not be eligible for second recruitment.

All follow-up visit procedures will be documented in the appropriate study forms. Clinical assessments and findings will also be documented in the child’s medical record, as appropriate.

#### Visits windows

- Visit + 3 days: same day.
- Visit at discharge: same day. Can be avoided if it is on the same day as visit + 15 days or + 30 days or in their windows.
- Visit + 15 days and + 30 days: window of 72 h
- Visit + 60 days: window of 1 week
- Visit + 90 days: window of 2 weeks
- Remaining visits can be completed within a 3-week window around the target date for subsequent visits.

### Sample size {14}

The study aims to enroll 624 HIV-infected infants altogether.

The sample size was estimated using WebPower R package (Ref: https://CRAN.R-project.org/package=WebPower). Sample size calculation was performed according to an 80% statistical power and a significance level of 0.05.

Several scenarios were considered according to survival (short-term and long-term) published data in HIV-infected children with pneumonia and TB. Baseline mortality was considered when only SoC had been assigned to the patients.

#### Baseline mortality estimations

##### Short-term mortality in HIV-infected children with pneumonia

A baseline of 35% 15-day mortality due to pneumonia is assumed for this calculation [6]. Out of this 35%, we estimated that 9% die due to TB, 24% die due to CMV pneumonia, and 2% due to other causes.

The calculation of the deaths related to TB is based on the prevalence of TB in children presenting with pneumonia (15%) [7, 11]. From this percentage, at least 75% of them are estimated to be unrecognized TB (11.25%) [13, 14]. Of them, 80% will die. This results in a baseline of 9% TB-related mortality.

The calculation of the deaths related to CMV is based on the prevalence of CMV in HIV-infected children with severe pneumonia (36–72%) [36, 37]. Without treatment against CMV or ART, CMV-related mortality varies between 50 and 85% without ART (average, 68%) [8, 9, 38]. Using a conservative 36% of prevalence, at least 24% of this baseline mortality is due to unrecognized, untreated CMV infection.

##### Long-term mortality in HIV-infected children with pneumonia

Long-term mortality was calculated according to a 10% reported mortality after discharge [1, 2]. In the 65% of survivors, this represents 6.5% of global mortality. A baseline of 41% of mortality at 1 year is assumed (short-term 35% + 6.5% later).

#### Benefits associated with empirical treatments

According to the literature, the benefits associated with both CMV and TB treatments have been considered for calculating the differences between basal mortality and treatment-reduced mortality.

##### The synergic benefit associated with both CMV and TB treatments

Despite the factorial design of this trial, the expected interaction effect is synergic. Usually, factorial designs with two drugs with a possible interaction need a high number of patients because the effect of combined interventions has a smaller effect than the two interventions added. However, in this case, the interaction expected is synergic. The two interventions act against different causes of death that can co-exist in the same patient. In addition, CMV has been shown to increase the pathogenesis of HIV and TB [39, 40]. The arm with two interventions is expected to have at least the same reduction of mortality as the two interventions added.

1. Short-term mortality combined treatment effect

The expected effect of TB-T in unrecognized TB mortality is a reduction of 90% mortality [13]. For 9% of mortality, the expected reduction of basal mortality with TB-T is 8% (90% of 9%).

The effect of valganciclovir in CMV pneumonia seems to be a reduction of 50% mortality at least [9, 38]. For 24% of CMV-attributed mortality, the expected reduction of basal mortality with valganciclovir is 12% (50% of 24%). Together, the expected reduction of 15-day mortality with TB-T and valganciclovir will be 20% (8% + 12%). From a basal 35% short-term mortality, the double treatment intervention will reduce mortality to 15% (35–20%). *According to this short-term mortality reduction effect, it is estimated that at least n = 156 patients will be enough to address this arm of the combined treatment effect in short-term mortality.*

2. Long-term mortality combined treatment effect

Bases on published data, the long-term mortality reduction of TB-T is at least 65% [34]. Among the 85% survivors after discharge, 10% mortality is expected (8.5%). This means a reduction of basal mortality of 5.5% (65% of 8.5%). Together, the expected reduction of long-term mortality will be 20% + 5.5% = 25.5%. From a basal 41% 1-year mortality, the double treatment intervention will reduce mortality to 15.5% (this is = 41–25.5%).

According to this 1-year mortality reduction effect and an estimated 5% of lost-to-follow-up, it is estimated that at least *n* = 110 patients will be enough to address this arm of combined treatment effect in long-term mortality*.*

##### The benefit associated with TB-T

1. Short-term mortality TB-T effect

The effect of TB-T in unrecognized TB mortality is a reduction of 90% mortality [13]. For 9% of mortality, the expected reduction of basal mortality with TB-T is 8% (90% of 9%).

From basal 35% 15-day mortality, TB-T will reduce mortality to 27% (35–8%). *According to this 15-day mortality reduction effect, it is estimated that at least n = 1052 patients will be needed to address this arm of TB-T effect in short-term mortality.*

2. Long-term mortality TB-T effect

The long-term mortality of 73% of survivors after discharge is reported to be 10% (7.3% of mortality) [1, 2]. The mortality reduction effect of TB-T has been shown to be 65%, so the expected reduction of basal mortality is 5% [34]. Altogether, a 1-year reduction from baseline mortality will be 8% + 5% = 13%. From a basal 41%, TB-T will reduce mortality to 28% (41–13%). *Taking into account 5% of lost-to-follow-up, it is estimated that at least n = 469 patients will be enough to address this arm of combined treatment effect in long-term mortality.*

##### The benefit associated with CMV treatment

1. Short-term mortality CMV treatment effect

The effect of valganciclovir in CMV pneumonia showed a reduction of 50% in mortality [13]. For 24% of CMV-attributed mortality, the expected reduction of basal mortality with valganciclovir is 12% (50% of 24%). From a basal 35% short-term mortality, valganciclovir will reduce mortality to 23% []. *According to the previous 15-day mortality reduction effect, it is estimated that at least n = 452 patients will be needed to address this arm of TB-T effect in short-term mortality.*

2. Long-term mortality CMV treatment effect

A benefit of valganciclovir on long-term mortality reduction is not expected. Long-term mortality of 31% is estimated according to short-term mortality (23%) plus the 10% mortality among the 77% survivors (8%). *According to this 1-year mortality reduction effect and an estimated 5% of lost-to-follow-up, it is calculated that at least n = 624 patients will be enough to address this arm of valganciclovir treatment effect in long-term mortality.*

Mortality and the prevalence of CMV and TB will be monitored during enrollment. Should new data arise relating to assumptions in this calculation, the sample size might be re-estimated at mid-enrollment interim analysis, after consultations with DSMB and Ethical Committees [41].

### Recruitment {15}

Enrollment is expected to be higher in the first 12 months and then decrease moderately due to improvement in PMTCT care. Patients will be enrolled by the research staff of each site. The estimation is enrolling two to five patients per month and per site, based on a previous inquiry to the sites.

Enrolment has been reinforced by adding new enrolling sites to the initially planned centers, and peripheral referral hospitals will be contacted to transfer potential participants to the enrolling sites. Staff will be trained through webinars about the trial pathways, the protocol details, potential and actual challenges, equipoise, and communication.

## Assignment of interventions: allocation

### Sequence generation {16a}

Each participant will be randomized in a 1:1:1:1 allocation ratio. Randomization will be stratified by center and clinical severity (presence of danger signs) to avoid that local practice at high-recruitment centers has a disproportionate effect on the results of the trial. An automatic randomization system will be designed in REDCap (Research Electronic Data Capture) using permuted blocks with variable block sizes and will be monitored by the CTU. The allocation sequence will be generated through computer-generated random numbers and a list of any factors for stratification. To reduce the predictability of a random sequence, details of blocking will be provided in a separate document that is unavailable to those who enroll participants or assign interventions.

### Concealment mechanism {16b}

Interventions will be assigned through a password-protected randomization service online. A telephone in the central team will be 24/7 available in case online assignation was not possible.

The generated randomization lists will be securely incorporated within the web trial database, and allocation concealed until the point of the next randomization. The designated members of the research staff at each site will be responsible for carrying out the randomization process using a secure electronic system within the trial database. Randomization will not take place until after informed consent has been given and the participant is ready to receive therapy.

A reliable manual back-up system will also be available. If the center’s internet connection is unavailable at the time of randomization, the screening details can be provided to staff at the FI+12-CTU by phone. At the CTU, staff will verify eligibility and perform the randomization using the online system. The details of the patient’s treatment allocation and the trial number will be notified to the local research team by phone within 1 h of the receipt of the randomization request.

### Implementation {16c}

The researcher will go to the electronic system REDCap to conduct the randomization and to get the participant identification number (ID) and allocation treatment arm. Randomization and enrollment occur at the same study visit, designated as day 0. Randomization is defined as the process of assigning a child to a study arm; assignments are computer-generated by the statisticians at central CTU. An automatic alert to the CTU will be sent to know a new patient has been enrolled. The researcher will get the randomization assignment after connecting online with the central CTU. An alternative method (phone) will be provided in case the internet is not working. Enrolled patients will be assigned to receive or not valganciclovir and/or TB-T. Once the patient is randomized, the researcher will open a patient file with the patient trial identification number.

## Assignment of interventions: Blinding

### Who will be blinded {17a}

The study is open. Data analysts are not blinded. The primary outcome—all-cause mortality and secondary outcomes are objective to reduce the risk of bias.

### Procedure for unblinding if needed {17b}

The trial is not blinded. Therefore, unblinding will not occur.

## Data collection and management

### Plans for assessment and collection of outcomes {18a}

A data management team (DMT) will be settled with one member of each site and members of the CTU, and they will create a Data Management Plan which will follow for data collection.

Source documents will include but are not limited to:

- Screening log
- Signed ICFs
- eCRF
- Hand-filled CRF
- Documentation of the comprehension checklist
- Visit documentation that includes dates of study visits
- Reported laboratory results
- Clinic notes
- Clinical record
- Prescription notes
- Pharmacy entry/exit documents
- Radiology images and reports

Laboratory results will be reported using paper or electronic reports following the habitual practice of each site. Those reported in paper will be included in the child’s medical records. Laboratory results that will be processed grouped in a second time will not be reported save those with relevant clinic implications. The incidental findings will be reported following the Tri-Council Policy (see next section “Incidental findings”). Site investigators will maintain and store securely, all source documents throughout the study. These documents will be retained for at least 5 years after the last child exits the study or more following the local Ethics Committee’s requests.

#### Case report forms

All study data will be collected by the clinical study staff using designated source documents. Electronic case report forms (eCRFs) will be developed for the collection of the trial’s data. Those centers without the capacity of using eCRF will use paper-based CRFs. Study data can be entered directly into the eCRFs during a study visit and uploaded into the server. In sites where paper-based CRFs will be used, data will be then entered into the electronic database as promptly as is feasible. After the introduction, data will be double-checked by a senior researcher when possible. CRFs and laboratory reports will be reviewed primarily by the site clinical team who are responsible for ensuring that they are accurate and complete. The eCRFs will be the data source since they are considered to have more information than the medical routine records. The eCRFs will be allocated in a secure server placed in the CTU site. Paper-based CRFs and other supporting documents (both electronic and paper-based) will be kept in a secure location and remain separate from participant identification information (name, address, etc.) to ensure confidentiality. GCP will be followed to ensure accurate, reliable, and consistent data collection.

#### Incidental findings

In the case of discovering incidental/unexpected findings, the Tri-Council Policy will be followed. The policy is based on evidence about the analytic and clinical validity of potential findings and their clinical significance, and careful consideration of the benefits and risks of disclosure.

The principal investigator (or representative) at the site will inform the caregiver by underlining the importance of the possibility of discovering incidental/unexpected findings.

The disclosing incidental/unexpected findings to study participant’s policy will follow the principles of:

- Respect for persons.
- Beneficence.
- Justice and fairness.
- Intellectual freedom and responsibility.

Wishes of participants regarding the disclosing of the incidental/unexpected findings will be asked in the ICF and respected in case if it were the case.

### Plans to promote participant retention and complete follow-up {18b}

Participants who do not attend any of the scheduled visits will be contacted telephonically by study personnel and through the community to assess the vital status and compliance with study interventions. All efforts will be done to assess the survival of the patients.

After discharge, TB-T will be provided up to the next visit, if randomized to TB-T, and after each visit up to the following visit. All patients will be scheduled equally for follow-up visits. Follow-up will end 12 months after the initial enrollment.

### Data management {19}

A centralized computerized database, managed by the CTU in REDCap, will be the central data repository for all sites participating in the trial. Each site participating in the trial will be responsible for data entry and first cleaning. Data collected electronically will be transferred from the sites to the CTU using an own secure server, allocated in Hospital 12 Octubre (Madrid) and maintained by the Clinical Trials and CTU in the Hospital 12 Octubre.

Sites will be able to self-edit, make online quality check reports, and be informed of the data collected during the clinical trial from their own site. Local investigators will have access during data collection time upon request and under justification to the complete pseudonymized database exportation. User-specific usernames and passwords are required to log onto the database. User rights will be provided to study staff, PIs, and coinvestigators at the level appropriate for each individual’s job description by the central CTU data managers.

After principal results, addressing primary and secondary objectives will be published, data repository will be publicly available by concrete permission of the CTU and data will be pseudo-anonymized and encrypted. Those interested in having the database or any of its subsets should provide a concrete research proposal that may be accepted under citation condition.

The local PIs will maintain, and store securely, complete, accurate, and current study records throughout the study. In accordance with regulations, study staff will retain all study records on-site for at least 5 years after study closure or more if required by the National Ethics Committee. Study records will not be destroyed before receiving approval for record destruction from the Sponsor. Applicable records include source documents, site registration documents and reports, ICFs, and notations of all contacts with the child.

### Confidentiality {27}

All study-related information will be stored securely at the sites. All participant paper information will be stored in locked file cabinets in areas with limited access provided by the local PIs. All laboratory specimens different from those included in the SoC, reports, process, and administrative forms will be identified only by an ID to maintain participant confidentiality. All records that contain names or other personal identifiers, such as locator forms and ICFs, will be stored separately from study records identified by the ID number. All local databases will be secured with encrypted and password-protected access systems. Forms, lists, logbooks, appointment books, and any other listings that link participant ID numbers to other identifying information will be stored in a separate, locked file in an area with limited access managed by the local PIs.

All HIV test results will be kept strictly confidential; all counseling will be conducted following local privacy customs.

Participants’ study information will not be released outside of the study consortium without the written permission of the participant. CTU will oversee the intra-study data sharing process, and they will be given access to the cleaned data sets. Project data sets will be housed in the CTU secure server allocated in Hospital 12 Octubre (Madrid, Spain), and all data sets will be encrypted and password protected. Project PIs will have direct access to their own site’s data sets and will have access to other sites’ data by justified request. To ensure confidentiality, data dispersed to study staff will be blinded to any identifying participant information. RedCap software implements an audit trail to ensure the data access tracking by the study personnel.

All records identifying the participant will be kept confidential and, to the extent permitted by the applicable laws and/or regulations, will not be made publicly available.

Procedures for data collection, storage, transfer, protection, and retention will be detailed in the trial Data Management Plan. Specifically, designed CRFs will be developed for collection of the trial’s data and will be designed in compliance with regulatory requirements for clinical trials, following the principles of ICH-GCP and the General Data Protection Regulation 2016/679. A centralized computerized database, managed by the CTU and allocated in the Hospital 12 Octubre (Madrid, Spain), will be the central data repository for all sites participating in the trial.

Patients will be assigned a trial identification number and will not be identified by their name.

The Sponsor will assure appropriate measures managing pseudo-anonymized and encrypted data. Apart from medical data, all ICFs will be stored and locked separately from the child’s trial folder with all other case record forms.

The child’s personal information will not be disclosed. The only people with access to the child’s personal information will be the employees of the hospital where the child is being looked after and Sponsor’s authorized Contract Research Organization (CRO). The child’s name will not appear in any information we or our partners publish about this study. Personal pseudo-anonymized information will be accessed by the study team members, the authorized representatives of the sponsor, SERMAS-Fundación 12 de Octubre, CRO, and the Ethics Committees but always without the name of the child. The data controller is SERMAS-FI+12. The processing of personal data is on the basis of the informed consent. The participant will have the right to access, rectify, or erase the child’s personal data; restrict the types of activities the research team can do with; object to using their and their child’s personal data for specific types of activities; or withdraw their consent.

No directly identifiable patient data will be held in the trial database, and the patient will be identified by a trial ID number. However, data that could be considered as indirectly identifiable (date of birth) or sensitive (date of medical events) will be handled within the guidelines of CTU standard procedures, which also details the sending and receiving of patient data under secure channels, either encrypted zip files or using inbuilt encryption software for email.

The retention period will be at least 5 years after the end of the trial or more if locally required by Ethics Committees after which time they will be securely destroyed or archived depending on national regulations. Paper documents will be stored locally in locked rooms. Paper documents will be also filed in a locked container or cabinet.

Similarly, all collected samples for only study purposes will be identified by the ID code. As specified above, samples will only be used for those purposes described explicitly in the ICF and information sheet in line with the approved protocol. Under no circumstances will those samples be stored or used for other purposes or further unplanned analyses not described in the protocol and ICF documents. In addition, the information sheet will also clearly state that samples will be transferred out of the country to both the EU and African countries for analysis. The requisite material transfer agreements will be obtained under national regulation.

Analyses will be conducted both locally and centrally. Samples will be analyzed at the recruiting centers, except CMV-PCR that will be analyzed in Zambia, PK samples that will be analyzed in the Netherlands, and MIAs samples that will be analyzed in Spain. After samples have been fully analyzed, they will either be discarded to a local storage facility at each of the site’s premises following the Trial Protocol.

### Plans for collection, laboratory evaluation, and storage of biological specimens for genetic or molecular analysis in this trial/future use {33}

#### Storage and analysis of clinical samples

The type of samples that will be collected, the storage, and the analysis procedures are summarized in Table 3. The visits in which these samples will be performed are described in Table 4. All the procedures will be described in the specific laboratory SOPs that will be approved by the Sponsor.

**Table 3:** Summarized of storage and analysis of clinical samples

Type of sample	Procedure	Minimum process volume required	Container	At time	Place of analysis	Storage	Shipment	Destruction^h^
Venous or finger/heel prick whole blood or plasma	HIV 1–2 RT-PCR POC automated nucleic acid testing platform (ALERE®)	0.3 ml	Cartridge/	Yes	Locally	No	No	According to manufacturer
			Capillary/					
			EDTA					
Venous whole blood	Total blood count Automatic coulter	0.5 ml	EDTA	Yes	Locally	No	No	
Venous whole blood	ALT, Creatinine, Glucose	0.5 ml	Dry tube	Yes	Locally	No	No	
	Automatic analyzer							
Venous whole blood	CD4 (absolute number and %)	0.2 ml	EDTA	Yes	Locally^e^	No	No	
Venous whole blood	HIV viral load	1 ml	EDTA	Yes	Locally^e^	No	No	
NPA^a^	Xpert MTB/RIF Ultra (TB PCR)	2–5 ml	Mucus extractor	Yes	Locally	No	No	According to manufacturer
Stools	Xpert MTB/RIF Ultra (TB PCR)	5 g	Stools container	Yes	Locally	No	No	According to manufacturer
Urine	Fujifilm TB LAM	5 ml	Urine container	Yes	Locally	No	No	According to manufacturer
Venous whole blood^f^	CMV viral load	1 ml	EDTA	No	Zambia	− 80 °C+/− 10 °C,	Only DNA ambient	
Saliva^f^	CMV viral load	Swab	Universal Transport Medium	No	Zambia	−80 °C+/− 10 °C	Only DNA ambient	
Sub-study and ancillary tests								
Venous whole blood^b^	PK1:t = 0, 2, 4, 6, 8, and 12 h after intake	1 ml each	Heparin	No	NTL	−80 °C+/− 10 °C	Dry ice	
Venous whole blood^b^	PK2: t = 0, 2, 4, 6, 8, 12/24 h after intake	0.5 ml each	EDTA	No	NTL	−80 °C+/− 10 °C	Dry ice	
Venous whole blood^b^	PK3: t = 2 and 5 after intake	1 ml each	Lithium heparin	No	NTL	−80 °C+/− 10 °C	Dry ice	
Venous whole blood^b^	PK4: t = 2	0.5 ml	Lithium heparin	No	NTL	−80 °C+/− 10 °C	Dry ice	
Blood, NPA, CSF and post-mortem tru-cut^c^	Advanced histopathological and microbiological^g^	10 ml, 2–5 ml, 10 ml, cylinders	EDTA	No	Spain	−80 °C+/− 10 °C	Dry ice	
			Dry tube					
			Dry tube					
NPA^d,f^	Cytokine levels	~ 2.5 ml (mucus + SPS)	Mucus extractor	No	Malawi	−80 °C+/− 10 °C	Dry ice	Following local guidelines
NPA^d,f^	CMV viral load, genotyping	~ 2.5 ml	Dry tube	No	Zambia	−80 °C+/− 10 °C	Dry ice	Following local guidelines

*Abbreviations*: *RT-PCR* real-time polymerase chain reaction, *ALT* alanine transaminase, *SPS* saline physiological solution, *PCR* polymerase chain reaction, *CMV* cytomegalovirus, *POC* point of care, *NPA* nasopharyngeal aspirate, *CSF* cerebrospinal fluid, *TB* tuberculosis, *EDTA* Ethylene-diamine tetra-acetic acid, *MTB/RIF* Mycobacterium tuberculosis DNA and resistance to rifampicin, *LAM* lipoarabinomannan, *PK* pharmacokinetics, *NTL* The Netherlands

^a^In the recruitment visit the NPAs will be performed in one action. In case the infant participates in the ancillary immune study the sample will be shared in 3 aliquots

^b^Sub-study performed only in Uganda, Zambia, and Zimbabwe

^c^Sub-study performed only in Mozambique and Zambia

^d^Ancillary study performed only in Blantyre and Lusaka Hospital in Zambia

^e^Some sites will send to the national referral laboratories

^f^Leftovers, in Zambia and Malawi, will be stored and later analyzed by molecular methods to identify potential differential causal pathogens and markers of bacterial and mycobacterial antibiotic resistance

^g^Bassat Q, Castillo P, Martínez MJ et al. Validity of a minimally invasive autopsy tool for the cause of death determination in pediatric deaths in Mozambique: An observational study. PLoS Med. 2017 Jun 20;14(6): e1002317

^h^According to trial SOPs for the sample, local guidelines and according protocol

**Table 4:** Summary of visits

Action		Staff member	Screening	Enrollment and randomization	Visit 1	Visit 2	Visit 3^*^	Visit 4	Visit 5	Visit 6	Visit 7	Visit 8
					3	discharge	15	30	60	90	180	360
Day			0	0	exact	exact	±3 days	±3 days	±7 days	±14 days	±21 days	±21 days
**SCREENING**												
Pre-screening and Screening log, inclusion assessment, Information, IC screening		Site investigators	X									
Screening for HIV, anemia and neutropenia		Site investigators	X									
**ENROLLMENT**												
Informed Consent		Site investigators		X								
Physical exam		Attending clinical staff		X	X	X	X	X	X	X	X	X
Ask for supplementary tests				X	X	X	X	X	X	X	X	X
Randomizaiton				X								
**INTERVENTION**												
Fixed-dose INH+RF+PZA prescription (2 mo)		Attending clinical staff		X	X	X	X	X				
Fixed-dose INH+RF prescription (4 mo)		Site investigators							X	X		
Ethambutol prescription (2 mo)		Site investigators		X	X	X	X	X				
Valganciclovir prescription (f15 days)		Attending clinical staff		X	X							
**STANDARD OF CARE**												
ART prescription		Attending clinical staff					X	X	X	X	X	X
Antimicrobials for pneumonia prescription		Attending clinical staff		X	X							
Cotrimoxazole prescription		Attending clinical staff		X	X	X	X					
Cotrimoxazole prophylaxis		Attending clinical staff						X	X	X	X	
Steroids prescription		Attending clinical staff		X	X	X	X					
**FOLLOW-UP**												
Clinical Research Form (CRF)		Attending clinical staff		X	X	X	X	X	X	X	X	X
Evaluation of adherence		Attending clinical staff		X	X	X	X	X	X	X	X	X
Serious adverse event form		Site investigators		X	X	X	X	X	X	X	X	X
Data entry		Data manager		X	X	X	X	X	X	X	X	X
**PHARMACY**												
Dispense of drugs, accountability, registry		Pharmacist		X	X	X	X	X	X	X	X	X
**SUPPLEMENTARY TESTS**												
**Image**												
Chest Radiography		Radiology		X								
**Labs**												
HIV-PCR		Phlebotomist/nurse	X		if needed							
Whole blood EDTA	CD count and %	Phlebotomist/nurse		X							X	X
	Full Blood Count	Phlebotomist/nurse	X			X	X	X			X	X
	Viral Load	Phlebotomist/nurse		X					X	X	X	X
Whole blood	Chemistry	Phlebotomist/nurse		X		X	X	X	X	X	X	X
**Labs (Microbiology)**												
Saliva	CMV	Phlebotomist/nurse		X			X					
Blood	CMV	Phlebotomist/nurse		X			X					
NPA	TBC routine	Phlebotomist/nurse		X								
Urine	TBC-LAM	Phlebotomist/nurse		X								
Faeces	TBC-PCR	Phlebotomist/nurse		X								
**Pharmacokinetics**												
Whole Blood	PK1	Phlebotomy/nurse						X				
Whole Blood	PK2	Phlebotomy/nurse							X			
Whole Blood	PK3	Phlebotomy/nurse			X							
Whole Blood	PK4	Phlebotomy/nurse						X		X	X	
Whole Blood	PK5	Phlebotomy/nurse						X		X	X	
**Immune Response ancillary study**												
NPA	Cytokine levels	Phlebotomist/nurse		X			X					


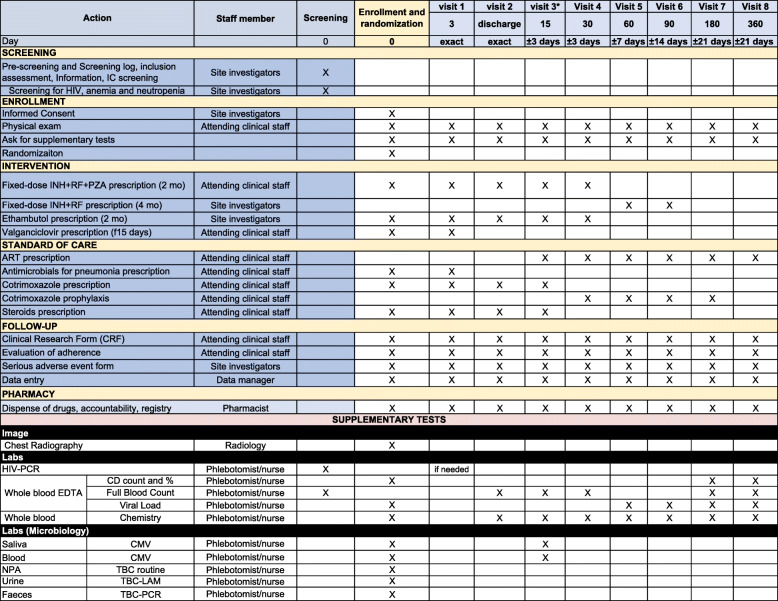


Minimal sample volumes that would be feasible for blood collection in infants will be drawn. The maximum amount of blood by weight will follow the WHO guidelines. If the total volume of blood could not be collected, the tests with implications for clinical care of the infant will be prioritized. Once those samples have been collected, any remaining blood will be analyzed following the order described in the correspondent SOP. All blood samples will be obtained through the least number of venipunctures.

The results of the samples that will be analyzed locally will be available at the usual time of each local laboratory and will be used to guide the infant’s clinical care. Those samples that will be analyzed in centralized laboratories will be shipped to the referral laboratory by an expert courier, preferably in a single shipment. Before shipping, they will be stored locally. The shipments will be made once all the recruited children have completed the follow-up visits from which these samples are required or before to assure the quality of the samples. The receiving laboratory will be responsible for arranging the transport of the samples in collaboration with the sites. A register of all the stored samples locally will be continually updated by the study staff of each site. The shipments of all the study samples will be registered in the trial master file.

From the main study, only the samples for CMV analysis (saliva and blood) will be analyzed outside of the local laboratories. This analysis will be centralized in Zambia (laboratory of the University Teaching Hospital in Lusaka) and will be done by researchers of the consortium. The other biological samples will be locally analyzed.

The samples from PK sub-studies will be analyzed in the Department of Pharmacy of Radboud University Medical Center, Nijmegen (Netherlands).

Once the samples arrive at the referral laboratories, they will generally be analyzed as soon as possible and always within the following 12 months. Once this analysis is performed, the unused remains will be destroyed as per the local country requirements and regulations relating to the disposal of biological research samples.

At the Zambian and Malawi sites, where sample volumes permit, leftover samples (blood, NPA and saliva) will be analyzed by molecular methods to identify potential differential causal pathogens and markers of bacterial and mycobacterial antibiotic resistance. The remaining samples locally analyzed will be destroyed as per the local country requirements and regulations relating to the disposal of biological research samples. All consumables, including extraction material, tubes, swabs, containers, and cartridges used for sample collection and local analysis, will be sourced locally by sites.

Different or additional samples to those described in the trial schedule could be performed to study infants at any time for the clinical management of the patient. Routine samples (e.g., blood cultures, malaria test) will be processed following the local procedure. Each local site will be responsible for ensuring that the samples are appropriately labeled in accordance with the trial procedures and comply with the 1998 Data Protection Act.

Biological samples collected from participants as part of this trial will be transported, stored, accessed, and processed in accordance with national guidelines/legislation relating to the use and storage of human tissue for research purposes and such activities shall at least meet the requirements as set out in the 2004 Human Tissue Act and the 2006 Human Tissue (Scotland) Act.

#### Full blood count, chemistry, HIV viral load, and CD4 samples

Samples will be processed locally using the ongoing laboratory methods in each site. Those samples that coincide in the calendar with the routine controls of the children will not be repeated but the results will be registered in the database and a copy of the source document will be kept.

## Statistical methods

### Statistical methods for primary and secondary outcomes {20a}

#### Primary analysis

As a primary design, the intent-to-treat (ITT) will be the main analysis strategy adopted, and per treatment analysis will be performed secondarily. ITT analysis will entail the comparison between randomization treatment arms *as-assigned*. An additional sensitivity analysis excluding early deaths ≤2 days after enrolment will be performed. According to clinical evidence, we estimate that 3% of randomized patients will meet this condition.

To describe the association between survival and the different effect of each arm treatment (valganciclovir and TB-T), raw logistic bivariate model and multivariable logistic model will be designed for dichotomous mortality (yes/no) and linear models for mortality rate (incidence rate density person-year). Odd ratios and 95% confidence interval will be displayed in association tables.

As a secondary analysis, the time-to-survival analysis will be performed to estimate HR for each randomization arm using flexible parametric models implemented in *flexsurv R package* (*Jackson C., 2016*). A flexible parametric model using restricted cubic splines (*Royston and Parmar, 2002*) will enable us to model the baseline hazard. HR and 95% confidence intervals will be assessed along with survival curves stratified by the randomization treatment arm.

#### Secondary analyses

As a secondary analysis, AEs and SAEs will be evaluated using an event-rate description (density incidence person-year) addressing the difference between treatment arms versus non-treatment. Chi-squared and Fisher exact test will be used for frequency comparisons. Unexpected adverse/serious reactions, the proportion of withdrawals of treatments, the proportion of treatments changed, and cumulative days of hospitalization will also be compared. To account for associations between valganciclovir and drug toxicity (number of AEs and SAEs), GLMM will be performed using a Poisson distribution with Laplace approximation implemented in *GLMMadaptive R package* (*Rizopoulos D., 2018*).

### Interim analyses {21b}

A specific interim analysis will be performed when 50% of study participants have been recruited, or halfway through the patient recruitment period, whichever occurs earlier. In the interim analysis, the primary endpoint mortality will be analyzed either in terms of density per person-year and survival time-to-event with right censoring.

Pre-specified interim analyses may be used for trial adaptations such as sample size re-estimation, alteration to the proportion of participants allocated to each study group, and changes to eligibility criteria. The trial will not be stopped in case of futility unless the DSMB, Trial Steering Committee (TSC), and EDCTP during the course of safety monitoring strongly advise otherwise. In this case, DSMB will discuss potential stopping for futility with the TSC. The TSC will decide on the continuation of the trial and will report to the Ethics Committees. We believe that this study is not susceptible to be stopped for futility where each arm indicates insufficient signs of the superiority of the treatment. The trial presents a valuable set of benefits (capacity building, educational aims, etc.) apart from the main hypothesis that justifies the continuation of the trial unless there is overwhelming evidence of harm symmetrical stopping boundary, either for efficacy or harm, will be adopted with a Fleming stopping rule of *p*-value < 0.001 in LTR test.

### Methods for additional analyses (e.g., subgroup analyses) {20b}

If it were the case that the model does not fit the assumptions and variance variability is high, generalized linear mixed model will be tested. Mixed effects linear models using smoothing splines will be tested including mortality rate (person-year) as the outcome variable and mixed effects logistic regression to dichotomous model mortality, in which fixed and random effects will be considered. Variance associated with random effects, fixed effects, and residual variance will be assessed, and model diagnostic plots will also be displayed. Bayesian models will also be tested to find the best-fitted model including Markov Chain Monte Carlo estimations implemented in MCMCglmm package (Hadfield JD., 2010). Variance associated with random effects, fixed effects, and residual variance will be assessed and model diagnostic plots will also be displayed.

### Methods in analysis to handle protocol non-adherence and any statistical methods to handle missing data {20c}

In order to avoid loss of information and statistical power in the association analysis, missing data will be imputed by means of multiple imputation chained equation method implemented in *MICE* R package. To prevent too many assumptions, only variables with less than 20% of missing information will be considered for imputation. To get a better understanding of the way missing data distribute among variables in the study, correlation matrixes, patching patterns, and box plot analyses will be performed. Sensitivity analysis will be performed to ensure low imputation derived bias, and an imputation appendix will be assessed in the study report. Finally, sensitivity analysis will then be undertaken to assess the robustness of the conclusions to assumptions regarding the missing data.

### Plans to give access to the full protocol, participant-level data, and statistical code {31c}

No later than 3 years after the collection of the 1-year post-randomization visits, the CTU will deliver a pseudo-anonymized data set and metadata to an appropriate data archive for sharing purposes unless specific national legislation from any of the sites impedes sharing open access of the data. In this case, the dataset of this site will not be released.

## Oversight and monitoring

### Composition of the coordinating center and trial steering committee {5d}

#### Trial steering committee (TSC)

The TSC will be composed by the Executive Committee and three independent members.

The Executive Committee is the primary governance body responsible for the strategic development plan and the ultimate decisions regarding critical issues affecting the whole project. It is composed by one deputy of each of the 15 partners and the Work Packages leaders and chaired by the EMPIRICAL Chief Investigator. The Executive Committee will meet face-to-face at least once per year during the project. The main decisions will be taken during these meetings. Decisions will be made by consensus whenever possible or by vote if needed. Each member of the Executive Committee will have one vote. Two-thirds of the members will be necessary to meet quorum. A simple majority of the attending members will be enough for decision adoption. In case of a tied vote, the Chief Investigator will have an additional vote. The Trial Management Group (see below) will participate in the Executive Committee meetings and will have a voice in the decision-making process. Other coinvestigators will be encouraged to participate.

The Executive Committee will be responsible for (i) development of the work-plan, (ii) approving the clinical trial protocol and all the related plan, (iii) assuring the fulfillment of the protocol, (iv) following up the implementation of the taken decisions, (v) supervising the use of project resources, and (vi) ensuring quality and standardization of research methodologies.

In order to accomplish these tasks, the Executive Committee will meet, at least every month, via teleconference.

The TSC will provide overall supervision for the trial and advice on its correct development with support from the independent members. EMPIRICAL TSC may decide to terminate the trial for any justifiable reason including the recommendation of the DSMB. The ultimate decision for the continuation of the trial lies within the TSC. TSC will meet at least once per year during the clinical trial.

#### Trial Management Group

It will be composed by the Project Management Team of the whole project that includes the Chief Investigator, the Scientific Coordinator, the Clinical Trial Coordinator, the Safety Coordinator and, and the Project Manager. The Project Management Team together with the Pharmacologist, the Data Manager, and the Statisticians will be the Trial Management Group. Their main activities will be:

- Study planning
- Organization of TSC and DSMB meetings
- Provide 6-monthly reports to DSMB meetings
- Provide annual risk report to be submitted to the Ethics Committees and regulatory authorities involves in the approval of the protocol
- Report serious unexpected suspected adverse events (SUSAR) to Medicines and Healthcare Products Regulatory Agencies
- Budget administration and contractual issues with individual centers
- Advice for principal investigators (PIs) of each recruitment site
- Central monitoring
- Assistance with correspondent regulatory agencies and Ethics Committees’ applications
- Data verification
- Randomization

The Trial Management Group will be supported by the Clinical Trial Unit of the Fundación para la Investigación Biomédica Hospital Universitario 12 de Octubre (F + 12-CTU). This CTU will act as the Clinical Trial Unit of the clinical trial.

### Composition of the data monitoring committee, its role, and reporting structure {21a}

Standard processes will be implemented to improve the accuracy of data entry and coding, including:

- Double check of data by local senior researchers for dead participants and those with SAES
- Central verification that the data are in the proper format (e.g., integer) or within an expected range of values
- Independent source document verification by an external Contract Research Organization (CRO) to identify missing or apparently erroneous values.

Site qualification and a pre-study visit will be performed by the CTU. A detailed site initiation visit with training will be performed at each study site by staff from CTU and the CRO. The site initiation visits will include training in the administration and side effects of study drugs, as well as the trial procedures.

#### Central monitoring

The FI+12-CTU will perform a secondary study database clean-up working closely with the participating sites to ensure that all collected data has been received and that all quality control checks were performed.

The DMT will develop a standard data definition table (DDT) or Masterfile which will be used to consolidate all sites’ data into a consistent format for storage in the central database. The DMT will maintain a specimen tracking process and link them to clinical data. The DMT will work closely with data managers from the sites to assure proper quality control checks are performed at the site level based on the central data editing plan. Data integrity will be validated by producing standard reports and distributing them to the sites on a routine basis. Standard processes will be implemented by local study personnel to enhance data quality and reduce bias by detecting and reducing the amount of missing or incomplete data, inaccuracies, and excessive variability in measurements.

Data entry and error checking will be completed in a timely manner to maximize the ability to resolve detected errors. DMT will perform an exhaustive quality control report checking for missing, unusual values, and clinical inconsistencies. Further outlier analysis will be performed in order to check possible data entry errors. If any such problems are identified, the site will be contacted and asked to verify or correct the entry. Changes will be made in the hard-copy CRF (if the site uses paper) and in the eCRF and entered into the database at the site. FI+12-CTU will also send reminders for any overdue and/or missing data with the regular inconsistency reports of errors. Each site will be responsible for developing a data entry error checking code, based on the standard data editing plan provided by the DMT. The sites will be responsible for following up on any queries produced by this code, to ensure the data are as complete and accurate as possible. All queries must be resolved within 2 weeks unless a specific date of resolution is requested by the DMT.

Quality controls will be conducted for the first five patients of each site and afterward, at least every 3 months. A single query table will be sent to each site in order to identify possible data entry errors. Changes will be made in the hard-copy CRF (if the site uses paper) and the eCRF and entered into the database at the site. FI+12-CTU will also send reminders for any overdue and/or missing data with the regular inconsistency reports of errors.

The investigator must assure that patients’ anonymity will be maintained and that their identities are protected from unauthorized parties. Patients will be assigned a trial identification number, and this will be used on CRFs; patients will not be identified by their names. The investigator will keep securely a patient trial register showing identification numbers, names, and dates of birth.

Data will be structured according to a final repository. A final repository will be chosen for anonymized data sharing, and transparency after the trial is closed, according to the funder (EDCTP) rules and recommendations, unless national laws impede it.

#### Site monitoring

Primary data management activities will be undertaken by the designated CRO. The on-site study data manager will oversee data-related procedures at the study site and will be supervised by the CRO data management staff.

The Sponsor and CRO monitors or their authorized representatives are responsible for contacting and visiting the study site for the purpose of inspecting the facilities and, upon request, inspecting the various records of the trial. These procedures will be also performed respecting participant’s confidentiality.

The site investigators will allow study monitors to inspect study facilities and documentation, e.g., ICFs, clinic and laboratory records, other source documents and CRFs, as well as observe the performance of study procedures. Medical records containing identifying information may be made available for review when the study is monitored by the Sponsor or an Authorized Regulatory Agency and the CRO. Direct access may include examining, analyzing, verifying, and reproducing any records and reports that are important to the evaluation of the study. Site visit logs will be maintained at the study site to document all visits.

#### Monitoring of accurate data recording in sites

Dedicated monitors from a CRO will visit all the clinical sites to validate and monitor data. They will conduct regular contact with sites and interim on-site monitoring visits. Site monitoring visits will be conducted to assess compliance with ICH-GCP guidelines. Study monitors will visit the site to:

- Verify compliance with human subjects and other research regulations and guidelines
- Assess adherence to the study protocol and study-specific SOP
- Confirm the quality and accuracy of the information collected at the study site and entered into the study database
- Assess the resolution of any past or ongoing issues identified at previous monitoring visits

The following data should be verifiable from source documents:

- Screening log
- Enrollment
- Review and verification of inclusion criteria
- All signed ICFs
- Eligibility and baseline values for all participants
- Pharmacy logs
- Review IMP accountability records (drug dispensed)
- Visits
- Dates of visits including dates when specimens were taken and processed in the laboratory
- All primary and secondary clinical endpoints
- Drug compliance data
- Concomitant medication
- Laboratory results
- Laboratory endpoints

Monitors will obtain corrections to CRFs/query resolution, maintain site training of personnel, and provide written site monitoring reports in every visit.

#### Visits to the sites

1. Starting visit
2. One visit 4 weeks after initiation of the trial or after first 5 patients enrolled.
3. Eight visits during enrollment

- Preparation: 8 h/visit
- Conduct visit: 10 h × 3 days / visit

4. Close-out visit

- Preparation: 8 h/visit
- Conduct visit: 12 h / visit

The CRFs of all patients enrolled will be reviewed at the first monitoring visit. In total, 100% of patients will be selected for review at subsequent visits. The monitors will require access to all patient medical records including, but not limited to, laboratory test results, and prescriptions. The investigators should work with the monitor to ensure that any problems detected are resolved.

Participating investigators should agree to allow trial-related monitoring, including audits, Ethics Committee review, and regulatory inspections by providing direct access to source data and documents as required. Permission for it will be asked to caregivers in the ICFs. Such information will be treated as strictly confidential and will not be made publicly available.

Deviations of the protocol and breaches will be reported by the site’s IP according to standardized SOP or by the CRO monitor in the visit’s reports that the CRO will do to all the centers in every visit.

#### Safety monitoring

This protocol has extensive safety monitoring in place. The study site investigators will be responsible for close safety monitoring of all children participating in the study, and for alerting the protocol team if unexpected concerns arise.

Safety coordinator together with the central data manager will perform biweekly safety data validation and include the queries in the data validation form. All queries must be resolved within 2 weeks unless a specific date of resolution is requested from the Safety Coordinator.

Each participating child will be evaluated by a study clinician at each study visit. If a child misses a study visit, home visits will be conducted by trained study staff to ensure clinical evaluation. Every effort will be made to trace all children in the study for the final outcome assessment. As needed, children in the study may be evaluated at interim visits and/or referred for additional care.

SAEs will also be regularly reviewed by the Safety Coordinator, and CTU compiled into reports for the DSMB every 6 months. The DSMB will be intimately involved in regular safety monitoring.

### Adverse event reporting and harms {22}

#### Pharmacovigilance

The principles ICH-GCP require that both investigators and the Sponsor follow specific procedures when notifying and reporting AEs in clinical trials.

##### Definitions

The definitions of the EU Directive 2001/20/EC Article 2 based on the principles of ICH-GCP applying to this trial protocol are given in Table 5.

**Table 5:** Safety definitions

Term	Definition
**Adverse event (AE)**	Any untoward medical occurrence in a patient or clinical trial subject to whom an IMP has been administered including occurrences that are not necessarily caused by or related to that product.
**Adverse reaction (AR)**	Any untoward and unintended response to an IMP related to any dose administered. Response to an IMP means that there is a reasonable possibility that there is a causal relationship between the AE and the medication, i.e., that relationship cannot be excluded.
**Unexpected adverse reaction (UAR)**	An AR in which nature or severity is not consistent with the information about the IMP in question set out in the SmPC or Investigator Brochure (IB) for that product.
**Serious adverse event (SAE) or serious adverse reaction (SAR) or suspected unexpected serious adverse reaction (SUSAR)**	Respectively any AE, AR or UAR that: •Results in death •Is life-threatening^a^ •Requires hospitalization or prolongation of existing hospitalization^b^ •Results in persistent or significant disability or incapacity •Consists of a congenital anomaly or birth defect •Is another important medical condition^c^

^a^The term life-threatening in the definition of a serious event refers to an event in which the patient is at risk of death at the time of the event; it does not refer to an event that hypothetically might cause death if it were more severe, for example, a silent myocardial infarction

^b^Hospitalization is defined as an inpatient admission, regardless of the length of stay, even if the hospitalization is a precautionary measure for continued observation. Hospitalizations for a pre-existing condition that has not worsened or for an elective procedure do not constitute an SAE.

^c^Medical judgment should be exercised in deciding whether an AE or AR is serious in other situations. The following should also be considered serious: important AEs or ARs that are not immediately life-threatening or do not result in death or hospitalization but may jeopardize the subject or may require intervention to prevent one of the other outcomes listed in the definition above; for example, a secondary malignancy, an allergic bronchospasm requiring intensive emergency treatment, seizures or blood dyscrasias that do not result in hospitalization or development of drug dependency

*Operational definitions for (S)AEs*

Adverse events

Adverse events include:

1. Exacerbation of a pre-existing illness
2. An increase in the frequency or intensity of a pre-existing episodic event or condition
3. A condition (even though it may have been present before the start of the trial) detected after randomization
4. Continuous and persistent disease or a symptom present at baseline that worsens following administration of the study treatment

Exempted adverse events

Adverse events do not include:

1. Medical or surgical procedures; the condition that leads to the procedure is the AE
2. Pre-existing disease or a condition present before treatment that does not worsen
3. Hospitalizations where no untoward or unintended response has occurred, e.g., elective cosmetic surgery, social admissions
4. Overdose of medication without signs or symptoms

Seriousness, severity, or grading of adverse events

When an AE or AR occurs, the investigator responsible for the care of the patient must first assess whether or not the event is serious using the definition given in Table 5 and the toxicity grading in Annex 1. The correspondent CRFs must be completed. If the event is *serious*, the IP of the site will notify it within *one working day* registering the AE in the electronic system and reporting it anonymized by mail to the CTU.

The severity of all AEs and/or ARs, serious and non-serious in this trial should be graded using the toxicity grading in Annex 1 (Toxicity Grading and Management). The IP of the site will notify all the *SAEs* within *one working day* and registering the AE in the specific CRF. Those centers using eCRF should upload to the electronic system of the filled eCRF and send a piece of advice by email. Those centers using paper CRF will fill and sign the CRF and will send a scanned copy by mail.

Causality

The investigator in each site must assess the causality of all AE and AR concerning the trial therapy using the standard definitions (Table 6). There are five categories: unrelated, unlikely, possible, probable, and definitely related. If the causality assessment is unrelated or unlikely to be related, the event is classified as an AE. If the causality is assessed as possible, probable, or definitely related, then the event is classified as an AR.

**Table 6:** Assigning Type of AE/AR through causality

Relationship	Description	AE type
Unrelated	There is no evidence of any causal relationship.	Unrelated AE
Unlikely	There is little evidence to suggest that there is a causal relationship (for example, the event did not occur within a reasonable time after the administration of the trial medication). There is another reasonable explanation for the event (for example, the patient’s clinical condition, and other concomitant treatment).	Unrelated AE
Possible	There is some evidence to suggest a causal relationship (for example, because the event occurs within a reasonable time after administration of the trial medication). However, the influence of other factors may have contributed to the event (for example, the patient’s clinical condition, and other concomitant treatments).	AR
Probable	There is evidence to suggest a causal relationship and the influence of other factors is unlikely.	AR
Definitely	There is clear evidence to suggest a causal relationship and other possible contributing factors can be ruled out.	AR

If an SAE is considered to be related to an IMP and the drug is stopped, or the dose modified, refer to section “Criteria for discontinuing or modifying allocated interventions {11b}”.

Expectedness

An unexpected AR is one not previously reported in the current SmPC or one that is more frequent or more severe than previously reported. If a SAR is assessed as being unexpected, it becomes a SUSAR. If there is at least a possible involvement of the IMP, the investigator in each site should make an initial assessment of the expectedness of the event. The CTU will have the final responsibility for the determination of expectedness (for reporting purposes), and this decision will be made based on the above definition and the information provided by the investigator.

Recording and reporting of (S)AEs, (S)ARs, and SUSARs

Deaths should be reported to Ethical Boards in the first 24 h and SUSAR in less than 7 days (Table 7). Other individual SAEs that are not SUSAR do not need to be reported in any time frame to the Ethical Boards unless the local Ethical Boards says differently.

**Table 7:** Recording and reporting guidelines for SAEs

	Individual SAE	Deaths	Aggregated SAEs
Who reports	Site Researcher PI	Site Researcher PI	CTU
How it is reported	AE CRF An email	AE CRF An email Collect all the related clinical information	Safety Report (SR) and Development Safety Update Reports (DSURs)
Timing	24 h since awareness	24 h since awareness	6-monthly (SR) Yearly (DSURs)
To Whom	CTU	CTU Ethical boards	SR: DSMB - 6 monthly DSUR: EDCTP, Regulatory authorities, Ethical boards - yearly
Who decides the relationship with IMP and expectedness	Site researcher opinion Confirm by CTU	Site researcher opinion Confirm by CTU DSMB	CTU DSMB

**Fig. 2:**
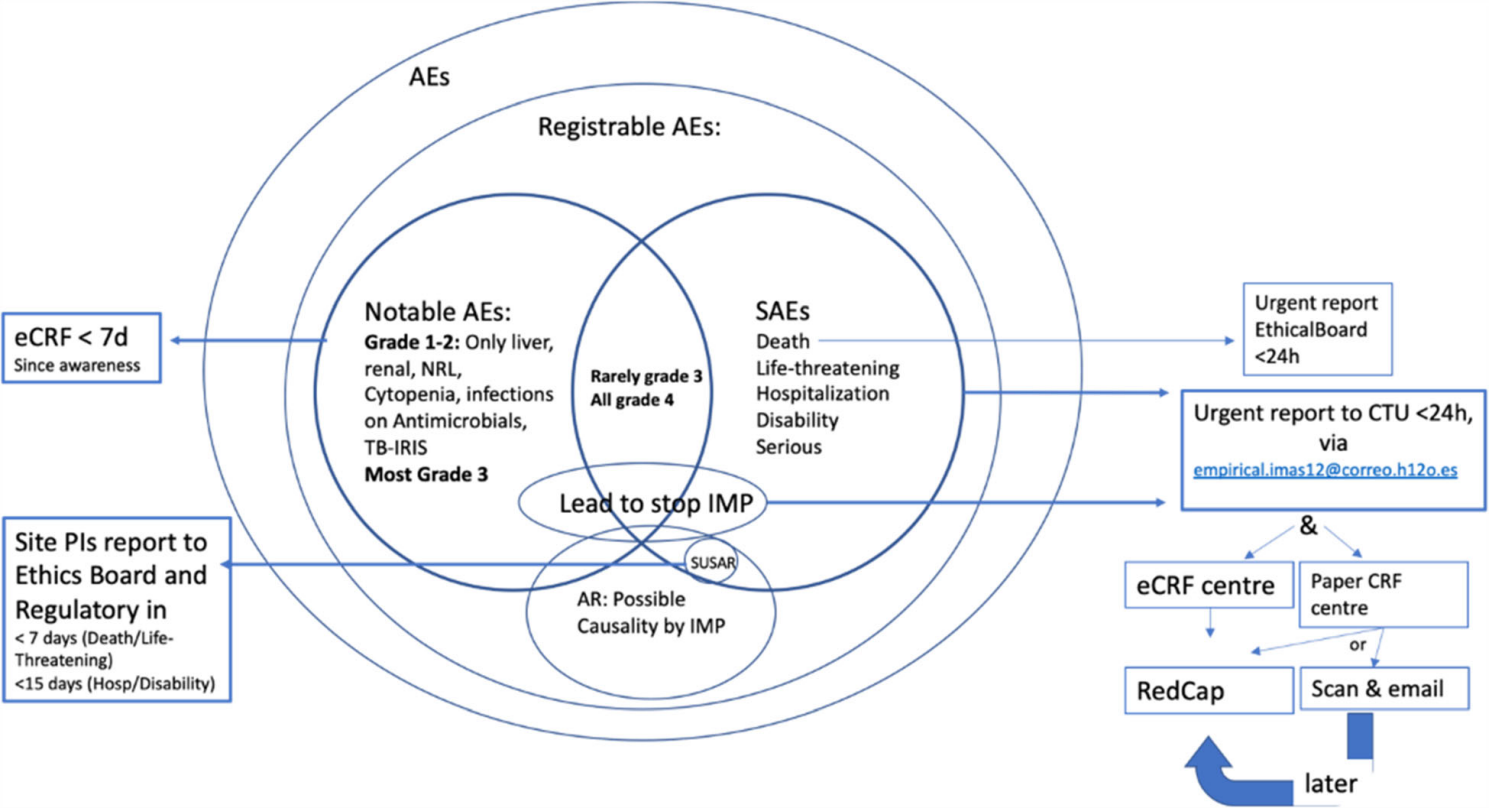
Scheme for reporting adverse events

As a general rule, any SUSAR should be reported to the National Drug Authority before 7 days (death or life threatening) or 15 days (hospitalization or disability) (Fig. 2).

Any SAEs and serious protocol deviations (not only deaths) should be reported to the Regulatory Authorities and Ethical Board within 24 h of becoming aware of the SAE and or protocol deviation. These should be adequately documented. Follow-up reports for the SAEs should be sent to the Regulatory Authorities within 7 days.

SAES

Investigators in sites should register and report to the CTU all the SAEs occurring during the trial and all the AEs that imply a change in the IMPs in the first working 24 h after their acknowledgment.

The period for which SAEs must be reported starts with randomization.

For ARs, SARs, and SUSARs start with the 1st IMP dose until the end of the trial. The report will be done by registering the event in the specific CRF and by email to the CTU. The CTU will also review all SAEs on the first working day, including death, and any AE in which the researcher thinks that IMP should be stopped/modified.

The CTU will use medical judgment in discussing with local PIs seriousness, causality, and whether the event/reaction was anticipated using the Reference Safety Information approved for the trial. The causality assessment given by the local investigator at the hospital cannot be overruled; in the case of disagreement, both opinions will be provided in any subsequent reports.

The CTU will review all reported AEs at least 6-monthly. Safety reports will be sent to the DSMB at least 6-monthly and will include a detailed analysis of study progress, data, and safety issues. The CTU will also keep all investigators informed of any safety issues that arise during the trial. CTU will prepare annual Development Safety Update Reports (DSURs) which will be submitted annually to the Competent Authorities and Ethics Committees in each country participating in the trial. Any subsequent events that may be attributed to treatment should also be reported to national reporting schemes where relevant.

The local PIs will record all SAEs. For each SAE, the following information will be collected:

- Full details in medical terms and case description
- Event duration (start and end dates, if applicable)
- Action taken
- Outcome
- Seriousness criteria
- Causality (i.e., relatedness to IMP), in the opinion of the investigator
- Whether the event would be considered anticipated
- Supplementary tests could be added

Notable AEs

Non-severe AEs will be registered only in the following cases: (i) all cases of liver injury, (ii) all suspected cases of renal injury, (iii) all cases of cytopenia, (iv) all neurological AEs, (v) all new infections requiring antimicrobial treatment, (vi) any AE that the investigator considers relevant, and (vii) any AE resulting in a change of IMPs (Table 8).

**Table 8:** Recording and reporting guidelines for notables AE/AR

	Individual AE	Aggregated AEs
**Who reports**	Site Researcher	CTU
**How it is reported**	AEs eCRF	Safety Report (SR) and Development Safety Update Reports (DSURs)
**Timing**	7 days since knowledge	6-monthly (SR) Yearly (DSURs)
**Whom**	Central CTU	SR: DSMB - 6 monthly DSUR: EDCTP, Regulatory authorities, Ethical boards - yearly

The period for which notable AEs must be reported starts with randomization.

##### Immune reconstitution inflammatory syndrome (IRIS)

Special attention will be paid to TB-related IRIS, as a secondary outcome. Suspected IRIS will be defined according to standard definition [42]. Any suspected IRIS will be registered. The previously described AEs will be considered *notable events*. Authorized study staff will register the notable events in the specific eCRF in the first 7 days since their knowledge. A not urgent report is needed for these notable events.

The recording and reporting guidelines are summarized in the following tables.

The specific CRFs of AEs must be completed by the investigator in charge of the site where they have occurred (with due care being paid to the grading, causality, and expectedness of the event as outlined above). Those considered an urgent reportable event (death, and any AE in which the researcher thinks that IMP should be stopped/modified) will be revised by the PI of the site. In the absence of the PI, the CRF should be reviewed and signed by another member/s of the site study staff that should be named on the Signature List and Delegation of Responsibilities Log.

In case it was determined that the event is an urgent reportable event, the information will be sent to the CTU using the online CRF in the next 24 h and or sending a scanned copy of the CRF by mail. The correspondent CRF should be subsequently checked in the first 24 working hours by the Safety team of the CTU, and extra information will be asked if needed, and the classification will be reevaluated. The initial report must be followed by detailed, written reports as appropriate.

Recommendations of stopping IMPs, possibilities of the reintroduction of IMPs, analytical controls needed, and additional treatments will be done according to specific SOP. The safety team of the CTU will be available to support these decisions in case they will be needed.

SUSAR

If an SAE is considered possibly, probably, or definitely related to an IMP, this SAE will be considered a SAR or a SUSAR, depending on expectedness (Table 9).

**Table 9:** Recording and reporting guidelines for suspected unexpected severe adverse reaction (SUSAR)

	Individual SUSAR	Aggregated AEs
Who reports	Site PI	CTU
How it is reported	Correspondent Ethics Committee Safety Report Form	Safety Report (SR) and Development Safety Update Reports (DSURs)
Timing	7 days since awareness (death and life-threatening) 15 days (hospitalization, disability)	6-monthly (SR) Yearly (DSURs)
Whom	Ethics Committees and Regulatory Authorities	SR: DSMB - 6 monthly DSUR: EDCTP, Regulatory authorities, Ethical boards - yearly

The Ethics Committees and Regulatory Authorities will be notified by the local IPs of all SUSARs with implications to the study within 7 days since awareness in case of death and life-threatening, and within 15 days in case of hospitalization or disability.

*Responsibilities*

1. Site PIs are responsible for reporting and registering urgently reported AEs. In the absence of the PI, the AE CRF should be reviewed and signed by another member/s of the site that should be named on the “Signature List and Delegation of Responsibilities Log”
2. Site PIs or delegate for this task is responsible for assigning type of AE/AR through causality
3. Site PIs or delegate for this task is responsible for making an initial expectedness of the SAEs
4. Research clinical staff are responsible for registering all the AEs following this SOP
5. Site PIs are responsible for reporting deaths to Ethical Boards
6. Site PIs are responsible for reporting SUSARs to Ethical Boards and Regulatory Boards but should discuss with CTU first.
7. CTU is responsible for reporting registrable AEs to DSMB.
8. Site PIs are responsible for notifying and send reports to the Ethics Committees and Regulatory Authorities all SAEs.
9. Site PIs are responsible to assign the type of AE/AR, and initial expectedness.

However, all efforts will be done to contact the CTU to agree on the early (< 24 h) report as much as possible. In each site, site PIs will ensure that notable AEs and ARs are recorded in line with the requirements of the protocol. Researchers will collect and verify all AEs, SAEs, SARs, and SUSARs according to this protocol onto a database.

*Notification of deaths*

All deaths will be reported to the Ethical Boards within the first working 24 h of becoming aware of the event.

*Pregnancy reporting*

This is not applicable.

*Overdose of IMPs*

All participants should be counseled about the importance of taking the medications as prescribed. Participants must be told to come to the clinic immediately if they take too many pills. Toxicity will be managed in both randomized groups according to standard clinical practice on a case-by-case basis in discussion with the safety coordinator of the CTU. Blood tests additional to those described in the trial schedule may be requested at any time for the clinical management of the patient.

*Reporting urgent safety measures*

If any urgent safety measures are taken, the Sponsor shall immediately and, in any event, no later than 3 days from the date the measures are taken, give written notice to the Medicines and Healthcare Products Regulatory Agency and the Ethics Committees of the measures taken and the circumstances giving rise to those measures.

*The type and duration of the follow-up of participants after adverse reactions*

Participants must be followed up until clinical recovery is complete and laboratory results have returned to normal or baseline, or until the event has stabilized. The AE form in CRFs will record the “Follow-up” AEs status, as information becomes available. Extra, annotated information, and/or copies of test results may be provided separately.

*Development safety update reports*

The CTU will provide (in addition to the expedited reporting above) DSURs every 12 months throughout the clinical trial, or as necessary, to the Competent Authority, and relevant the Ethics Committees.

### Frequency and plans for auditing trial conduct {23}

Participating sites will maintain appropriate medical and research records for this trial, in compliance with ICH-GCP and General Data Protection Regulation (GDPR), regulatory, sponsoring organization, and institutional requirements for the protection of personal data and confidentiality of children. The site will permit authorized representatives of the Sponsor and Regulatory Agencies to examine (and when required by applicable law to copy) clinical records for the purposes of quality assurance reviews, audits, and evaluation of the study safety and progress. User rights will be provided to study staff, PIs, and coinvestigators at the level appropriate for each individual’s job description.

#### Data and Safety Monitoring Board (DSMB)

There will be an independent advisory committee created to guide and advise the TSC. Its primary responsibilities will be to periodically review and evaluate the accumulated study data for participant safety. The DSMB will receive reports about serious adverse events (SAEs) every 6 months, and it will make recommendations to the Executive Committee and TSC regarding the continuation or modification of the trial. The DSMB will meet within 6 months after the trial opens face-to-face or online; in general, face-to-face meetings will be planned annually, but the frequency will be determined by the Executive Committee and could be more frequent if deemed necessary.

Members of the DSMB will include one independent clinical scientist with expertise in the study drugs, one ethics expert, and one independent statistician. Reports to the DSMB will be coordinated by the safety coordinator and performed by the trial statistician. Communications from the DSMB will require the participation of all three members

### Plans for communicating important protocol amendments to relevant parties (e.g., trial participants, ethical committees) {25}

Under the Medicines for Human Use (Clinical Trials) Regulations 2004, the Directive 2001/20/EC and Clinical Trial Regulation EU No. 536/2014, the sponsor may make a substantial amendment at any time during a trial. Substantial amendments will not be implemented at the site until the concerned Ethics Committee has approved, except where necessary to eliminate an immediate hazard to a trial patient or when the change(s) involves only logistical or administrative aspects of the trial.

If applicable, other country-specific specialist review bodies will be notified about substantial amendments in case the amendment affects their opinion of the trial.

Any other deviation from the protocol that has not been approved by the Sponsor and the Ethics Committee could result in discontinuation from the study of the center involved.

A substantial amendment is defined as an amendment to the terms of the application, or to the protocol or any other supporting documentation, that is likely to affect to a significant degree:

- The safety or physical or mental integrity of the infants of the study
- The scientific value of the study
- The quality and safety of study treatment
- The conductor management of the study

In these cases, a written amendment will follow the regulations in place. All substantial amendments will be notified to the Ethics Committee and the Competent Authority. Non-substantial amendments will not be notified to the Ethics Committee and the Competent Authority but will be recorded and filed by the Sponsor.

All investigators participating in the study must be aware of any protocol amendments and must respect their content.

### Dissemination plans {31a}

The Executive Committee will review all publications following the guidelines given below.

#### Data analysis and release of results

The scientific integrity of the project requires that the data from all the sites be analyzed study-wide and reported as such. Thus, an individual center is not expected to report the data collected from its center alone. All presentations and publications are expected to protect the integrity of the major objective(s) of the study.

#### Review process

Each paper or abstract must be submitted to the Executive Committee for review of its appropriateness and scientific merit before submission, giving enough time for review of a manuscript or journal article for review of any poster, presentation, abstract, or other written or oral material derived from the research. The Executive Committee may recommend changes to the authors and will finally submit its recommendations.

#### Primary outcome papers

EDCTP expects that grant holders will disclose the summary results of the study within 12 months from primary study completion (the last visit of the last subject for the collection of data on the primary outcome). The primary outcome papers will be those that present outcome data.

The authorship of the publications will be decided by the Executive Committee and by EDCTP rules.

Publication in a journal is expected within 24 months from study completion, according to EDCTP rules. The Trial identification number or registry identifier code should be included in all publications of clinical trials and should be provided as part of the abstract to PubMed and other bibliographic search databases for easy linking of trial-related publications with clinical trial registry site records. This is essential for linking journal publications with registry records.

Beneficiaries must acknowledge EDCTP funding as listed in the Grant Agreement. The beneficiaries must express the contribution in each publication by acknowledgment or co-authorship according to EDCTP rules.

The final report to EDCTP must include a report on the status of posting results in the study registry (including timelines when the final posting of results is scheduled after the end of the funding period).

Research results should be reported following the recommendations of the CONSORT Statement or an alternative reporting guideline appropriate to the study design (see the EQUATOR Network).

#### Other study papers, abstracts, and presentations

All analysis other than those designated as “Primary Outcome” fall within this category. This includes reports addressing in detail one aspect of EMPIRICAL, but in which the data are derived from the entire study and reports of data derived from a subset of centers by members of the EMPIRICAL (e.g., sub-studies or ancillary studies), or reports of investigations initiated outside of the EMPIRICAL, but using data or samples collected by EMPIRICAL. All papers and abstracts must be approved by the Executive Committee before they are submitted. Authorship will include the quote “from the EMPIRICAL Clinical Trial Group.”

The EMPIRICAL participant box will list all professionals that have participated in the clinical trial for a minimum of 1 year.

#### Close-out procedures

The clinical trial will terminate at the planned target of 12 months after the last participant has been randomized or at an earlier or later date if the circumstances warrant. Regardless of the timing and circumstances of the end of the study, close-out will proceed in two stages:

• Interim period for analysis and documentation of study results

• Debriefing of participants and dissemination of study results

1. Interim: Every attempt will be made to reduce to an absolute minimum the interval between the completion of data collection and the release of the study results. We expect to take about 6 months to compile the final results paper for an appropriate journal.
2. Reporting of study results: The study results will be released to the participating physicians, referring physicians, patients, and the general medical community.

#### Data sharing statement

No later than 3 years after the collection of the 1-year post-randomization visits, the CTU will deliver a pseudo-anonymized data set and metadata to an appropriate data archive for sharing purposes unless specific national legislation from any of the sites impedes sharing open access of the data. In this case, the dataset of this site will not be released,

## Discussion

During the implementation and start of the trial, the COVID-19 pandemic occurred. The pandemic led to partial or complete lockdown during different periods of 2020 and 2021 in all countries involved in the trial, especially impacting the beginning of the enrolment. Enrolment was 3 months delayed or halted in all the sites and was reinitiated at a slower pace than expected.

A specific plan was implemented in response to the COVID-19 outbreak, including the safety of the participants in the clinical trial and their families, the security of the study staff and other EMPIRICAL workers, compliance with country-specific national recommendations and guidelines, achievement of the main objective of the study as outlined in the protocol, and feasibility of implementation of the project as described, drug shipment, testing of enrolled participants, implications of positive COVID-19 results, the effect of COVID-19 in mortality, and follow-up of the patients. Monitoring visits are being done remotely during the time of limitations to travels at all the sites. Given the impact of COVID-19 on the enrolment, an extension of the trial was requested to the funders, sponsor, ethical boards, and regulatory boards.

## Trial status

Version 2.0, January 2021

Recruitment started on March 25th, 2020.

Approximate date for completion: January, 31st, 2024.

## Supplementary Information

**Additional file 1.**

## Abbreviations

3TC: Lamivudine
ABC: Abacavir
AE: Adverse event
ALT: Alanine transaminase
AR: Adverse reaction
ART: Antiretroviral treatment
AZT: Zidovudine
CMV: Cytomegalovirus
CRF: Case Report Form
CRO: Contract Research Organization
CTU: Clinical trials unit
DMT: Data Management Team
DNA: Deoxyribonucleic acid
DSMB: Data safety monitoring board
DSURs: Development Safety Update Reports
EC: European Commission
eCRF: Electronic Case Report Form
EMA: European Medicines Agency
EU: European Union
EudraCT: European Clinical Trials Database
FDA: Food and Drug Administration
FDC: Fixed-dose combination
FI+12: Fundación para la Investigación Biomédica Hospital Universitario 12 de Octubre
FI+12-CTU: Fundación para la Investigación Biomédica Hospital Universitario 12 de Octubre Clinical Trial Unit
GCP: Good Clinical Practice
GLMM: Generalized linear mixed model
GLP: Good Laboratory Practice
HIV: Human immunodeficiency virus
HR: Hazard ratio
ICF: Informed Consent Form
ICH: International Conference on Harmonization of technical requirements for registration of pharmaceuticals for human use.
IMP: Investigational Medicinal Product
IPT: Isoniazid preventive therapy
IRIS: Immune reconstitution inflammatory syndrome
ITT: Intention to treat
LAR: Legally authorized representative
LPV/RTV: Lopinavir-ritonavir
MIA: Minimally invasive autopsy
PMTCT: Prevention of mother-to-child transmission
NPA: Nasopharyngeal aspirate
NRTI: Nucleoside reverse transcriptase inhibitor
NVP: Nevirapine
PCP: Pneumonia by *P. carinii*
PI: Principal Investigator
RNA: Ribonucleic acid
SAE: Serious adverse event
SAP: Statistical analysis plan
SAR: Serious adverse reaction
SmPC: Summary of Product Characteristics
SoC: Standard of care
SOP: Standard operational procedure
SUSAR: Suspected unexpected serious adverse reaction
TB: Tuberculosis
TB-T: Tuberculosis treatment
TSC: Trial Steering Committee
UAR: Unexpected adverse reaction
WHO: World Health Organization
