# Supplementary material for: Empirical treatment against cytomegalovirus and tuberculosis in HIV-infected infants with severe pneumonia: study protocol for a multicenter, open-label randomized controlled clinical trial
Source: Trials. 2022 Jun 27;23:531. doi: 10.1186/s13063-022-06203-1 (PMC9235074; doi:10.1186/s13063-022-06203-1)

Annex 10: **Definitions of TB cases for Research Evaluation and Reporting Purposes.** Reference: 1: Graham SM, Cuevas LE, Jean-Philippe P, Browning R, Casenghi M, Detjen AK, Gnanashanmugam D, Hesseling AC, Kampmann B, Mandalakas A, Marais BJ, Schito M, Spiegel HM, Starke JR, Worrell C, Zar HJ. Clinical Case Definitions for Classification of Intrathoracic Tuberculosis in Children: An Update. Clin Infect Dis. 2015 Oct 15;61Suppl 3(Suppl 3):S179-87. doi: 10.1093/cid/civ581. PMID: 26409281; PMCID: PMC4583568.

**Definitions of TB cases for Research Evaluation and Reporting Purposes**


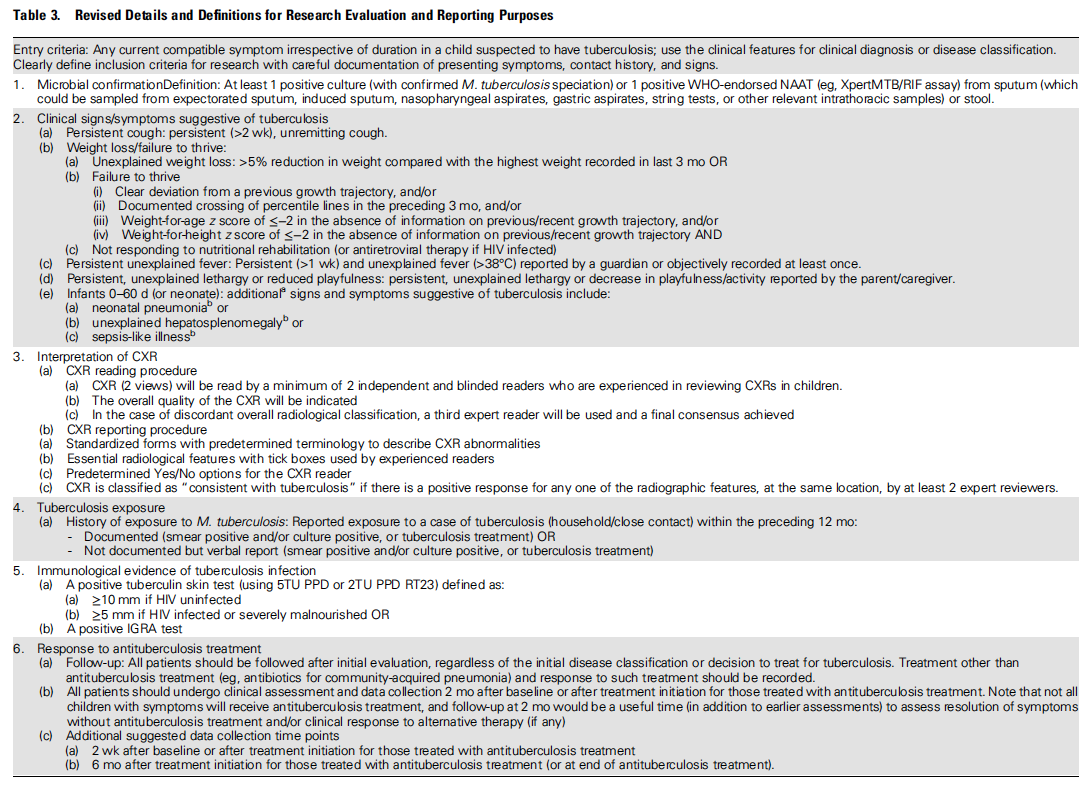


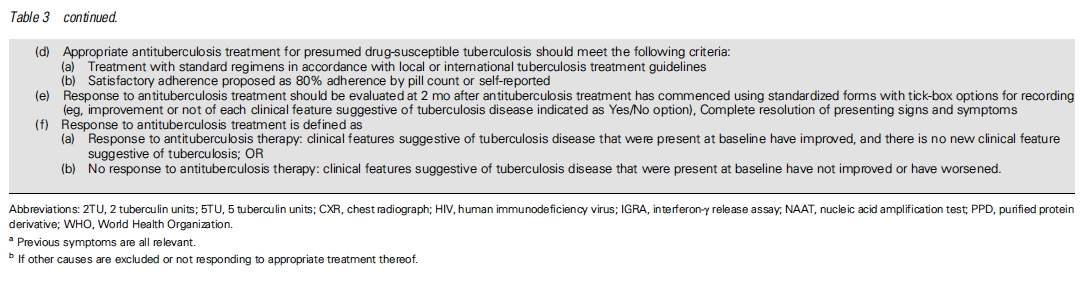

Supplement: Supplementary file 1 — Additional file 1. [file 13063_2022_6203_MOESM1_ESM.docx]
